# Efficacy of ANTHRASIL (Anthrax Immune Globulin Intravenous (Human)) in rabbit and nonhuman primate models of inhalational anthrax: Data supporting approval under animal rule

**DOI:** 10.1371/journal.pone.0283164

**Published:** 2023-03-17

**Authors:** Srinivas Kammanadiminti, Jason Comer, Gabriel Meister, Trevor Carnelley, Derek Toth, Shantha Kodihalli

**Affiliations:** 1 Emergent BioSolutions Canada (Previously Cangene Corporation), Winnipeg, MB, Canada; 2 Battelle Biomedical Research Center, Columbus, Ohio, United States of America; The University of Kansas, UNITED STATES

## Abstract

To meet the requirements of the Animal Rule, the efficacy of monotherapy with ANTHRASIL^®^ (Anthrax Immune Globulin Intravenous (Human)) for inhalational anthrax was evaluated in blinded studies using rabbit and nonhuman primate models. Animals in both studies were randomized to treatment groups exposed to ~ 200 LD_50_ *Bacillus anthracis* (Ames strain) spores by the aerosol route to induce inhalational anthrax. Rabbits (N = 50/group) were treated with either 15 U/kg ANTHRASIL or a volume-matching dose of IGIV after disease onset as determined by the detection of bacterial toxin in the blood. At the end of the study, survival rates were 2% (1 of 48) in the IGIV control group, and 26% (13 of 50) in the ANTHRASIL-treated group (p = 0.0009). Similarly, ANTHRASIL was effective in cynomolgus monkeys (N = 16/group) when administered therapeutically after the onset of toxemia, with 6% survival in the IGIV control and a dose-related increase in survival of 36%, 43%, and 70% with 7.5, 15 or 30 U/kg doses of ANTHRASIL, respectively. These studies formed the basis for approval of ANTHRASIL by FDA under the Animal Rule.

## Introduction

*Bacillus anthracis*, the etiologic agent of anthrax, is a gram-positive, rod-shaped, facultative anaerobic, spore-forming bacterium that can cause human disease via the gastrointestinal, cutaneous, or inhalational (pulmonary) routes.

Inhalational anthrax has historically been rare, with 21 cases reported in the United States from 1900 to 2001. However, this form of *B*. *anthracis* infection has been highly lethal with > 86% mortality in the absence of treatment. Aggressive treatment reduced mortality in cases associated with the exposure to *B*. *anthracis* spores by mail within the United States in 2001 to 45% (5 of 11 cases) [1]. Furthermore, an accidental release of *B*. *anthracis* spores from a Soviet bioweapons production facility in Sverdlovsk in 1979 resulted in at least 66 fatalities, with cases of both human and animal anthrax reported several kilometers downwind [2, 3].

Inhalational *anthrax* is characterized by a dose-dependent incubation period, of one to five or more days [4, 5]. Data from modeling based on anthrax cases suggested an incubation period of 4–15 days [6, 7]. Initial nonspecific symptoms such as malaise, headache, fever, nausea, and vomiting are followed by a sudden onset of respiratory distress with dyspnea, stridor, cyanosis, and chest pain leading to shock and death [1, 8–10]. Mortality caused by *B*. *anthracis* is predominantly due to three well-characterized virulence factors: lethal factor (LF), edema factor (EF), and protective antigen (PA) [11, 12]. Both LF and EF interact separately with PA to form two interlinked toxins: lethal toxin (LT) is a combination of PA and LF, and edema toxin (ET) is a combination of PA and EF [13, 14]. LT is the predominant cause of severe disease and death following inhalational *B*. *anthracis* spore exposure [15–17].

Vaccination is an effective prophylactic therapy for inhalational anthrax both in humans and animals; however, vaccination requires multiple doses, and the immunity conferred is not durable [18]. Currently, the United States Center for Disease Control (CDC) recommends vaccination only for those at risk of exposure to *B*. *anthracis*, such as military personnel, laboratory personnel who work with *B*. *anthracis*, and people who handle potentially infected animals [19]. Since vaccination does not elicit immediate protection against anthrax and there is an increased threat of *B*. *anthracis* as a bioweapon, monoclonal and polyclonal antibodies to the bacterial toxins have been developed as adjunct therapies for antimicrobials [20, 21]. Because of the high mortality rate of systemic anthrax and the seemingly low risk associated with antitoxin treatment, current CDC guidelines recommend using these antitoxins in conjunction with antibiotic therapy in patients suspected of having systemic *B*. *anthracis* infection [22].

To date, three antitoxins have been developed and approved by the United States Food and Drug Administration (FDA). Raxibacumab [23] and obiltoxaximab [21] were approved in 2012 and 2016, respectively, and are monoclonal antibodies binding to the domain IV of PA [24, 25]. ANTHRASIL^®^ (Anthrax Immune Globulin Intravenous (Human)) was approved in 2015 and is a polyclonal product derived from the plasma of donors vaccinated against *B*. *anthracis* [20]. All three antitoxins were approved based on the FDA’s Animal Rule [26], which allows licensure following demonstration of efficacy in animal models when conventional human clinical trials for efficacy are not feasible on ethical grounds. The primary animal models used to evaluate the efficacy of the vaccine and drug candidates against aerosolized *B*. *anthracis* have been rabbits and nonhuman primates (NHP) [22, 27–42].

Here we describe studies in rabbits and NHPs to demonstrate the therapeutic efficacy of ANTHRASIL against inhalational anthrax as part of the ANTHRASIL licensure program.

## Materials and methods

### Animal study designs

Two studies were conducted: one in New Zealand white rabbits and one in cynomolgus macaques. Both were randomized, blinded and placebo-controlled studies conducted in compliance with Good Laboratory Practices (GLP, 21 CFR 58) [43]. In both studies, animals were treated with ANTHRASIL or Immune Globulin Intravenous (IGIV) by intravenous infusion after real-time detection of circulation toxemia (PA antigen) in blood following lethal aerosol challenge with *B*. *anthracis* (Ames strain) spores. An additional group of untreated controls was used in the rabbit study to confirm the lethality of the aerosol challenge and obtain model development data. In accordance with the Animal Rule requirements, survival was the primary measure of efficacy for both studies. Additional metrics assessed included toxemia, bacteremia, hematology, clinical presentation and pathology. An individual animal is an experimental unit. Survival was the primary endpoint for both studies.

The rabbit study included an IGIV (control) group, an ANTHRASIL-treated group (N = 50/group) and an untreated group of 10 animals. A sample size of 50 animals per group was selected to ensure that at least 40 animals per group would be bacteremic prior to treatment, providing more than 80% power to detect a difference between the group survival rates of 0% and 20% in IGIV and ANTHRASIL groups, respectively at a significance level of 5%. For the primary efficacy analysis, the test statistic used was the Fisher’s Exact test; as this study was the pivotal study for licensure, a more stringent two-tailed version of this test was used.

The NHP study included four groups that received either IGIV or one of three doses of ANTHRASIL (N = 16/group). A sample size of 16 animals per group provided 80% power at a significance level of 5%, assuming a 15% survival rate in the control group and a 65% survival rate in the treatment group. This sample size also provides 80% power assuming a 10% survival rate in the control group and a 55% survival rate in the treatment group. Power calculations were performed using StatXact (version 8.0). For the primary efficacy analysis, a comparison of the survival rates was performed between each treatment group (groups 2, 3 and 4) and the control group (group 1) using a Fisher’s Exact test. As this study was exploratory in nature and a survival rate of less than 15% in the treatment group was not reasonably expected, the less stringent one-tailed version of this test was used.

For statistical analysis, the intent-to-treat (ITT) set of animals included those animals that were confirmed to be toxemic prior to treatment and received a full dose of either placebo or ANTHRASIL. The modified intent to treat (MITT) set of animals included those in the ITT group that were confirmed to be bacteremic at any time point prior to the treatment. Any animal that died due to non-anthrax-related causes was excluded from the analysis.

### Test facilities

Animal studies and all analyses including histopathology were performed by Battelle, West Jefferson, OH, USA. Toxin Neutralization Assays (TNA) and PA-Enzyme- Linked Immuno-Sorbent Assays (ELISA) were performed by Emergent BioSolutions, Winnipeg, Canada.

### Ethics statement

Animal studies were conducted in a Biosafety Level 3 facility at Battelle, West Jefferson, OH, USA. All studies were approved by the Battelle Biosafety Committee and the Institutional Animal Care and Use Committee (IACUC) of Battelle, which adhere to the Animal Welfare Act and the Guide for the Care and Use of Laboratory Animals (Permit Number: A3034-01).

### Animal models

#### Rabbits

A total of 110 (55 male, 55 female) specific-pathogen-free New Zealand White (NZW) rabbits (*Oryctolagus cuniculus*) with surgically implanted venous access ports were purchased from Covance Laboratories (Denver, PA, USA). The patency of catheter lines and venous access ports (VAPs) was maintained regularly. Females were nulliparous and non-pregnant. Following a quarantine period of one week, rabbits that were in good health and free of malformations were released from quarantine for placement on the study. Rabbits were singly housed and weighed 2.3–3.5 kg on the day of challenge. Based on body weights, the ages of the rabbits used were estimated to be approximately 8–10 weeks.

#### Nonhuman primates

A total of 64 (32 male, 32 female) cynomolgus monkeys (*Macaca fascicularis*) of Vietnamese origin from Covance laboratories (now Envigo, Alice, TX, USA) were randomized into four treatment groups. Prior to the start of the study, all Nonhuman primates (NHPs) were surgically implanted with VAPs and TA-D70 DSI radio-telemetry transmitters (Data Sciences International, St. Paul, MN, USA). Animals were single-housed before surgery and then during post-surgical recovery and weighed 2.1 to 3.1 kg prior to aerosol challenge. Based on body weights, the ages of the NHPs used were estimated to be approximately 2–4 years.

Nonhuman primates were acclimated to pole/collar restraint during quarantine. Prior to receipt at Battelle, all NHPs were screened and proved negative for *Mycobacterium tuberculosis*, antibody negative by serological assay for Herpes B, Simian Retrovirus (SRV), and Simian T-Cell Leukemia Virus (STLV)-1, and negative by PCR for SRV1 and 2. During quarantine, animals received a physical examination by a veterinarian, a complete blood count and clinical chemistry analysis, a fecal float test and three additional tuberculosis skin tests prior to inclusion in the study. Following the first observation of any abnormality, animals were closely monitored so as to make a judgment on euthanasia. Monkeys that developed non-study -related illnesses, such as physical injuries, were evaluated by a veterinarian for determination of treatment or disposition.

### Exposure to *Bacillus anthracis* spores

All aerosol challenges occurred with a well-characterized, single lot of *B*. *anthracis* (Ames strain) spores [44]. Characterization involved percentage encapsulation (pXO1/pXO2 gene expression), colony morphology on blood agar, microscopic observation for culture purity, percentage spore refractility, Guinea pig LD_50_ and spray factor.

A modified type three-jet Collison nebulizer (BGI, Waltham, MA, USA) was used to generate a controlled delivery of aerosolized *B*. *anthracis* Ames spores from a liquid suspension. The aerosol particle size was determined during each exposure using an Aerosol Particle Sizer (APS) spectrometer (Model 3321, TSI Inc., Shoreview, MN, USA). For each animal, the aerosol concentration of spores to which they were exposed was calculated by plating dilutions of a sample collected from an all glass impinger (Ace Glass, Inc., Model 7541), which sampled the aerosol environment during the entire exposure period. Inhaled doses were determined from the individual real-time plethysmography data and aerosol spore concentration as described previously [27, 36].

Rabbits were exposed muzzle-only to aerosol challenge, whereas each NHP was anesthetized prior to challenge, placed into a plethysmography chamber, and put in a Class III cabinet system for spore exposure. The targeted aerosol challenge dose was 200 LD_50_ (2.1 × 10^7^ CFU for rabbit [45] and 1.2 x 10^7^ CFU for NHP [36, 46]).

### ANTHRASIL and placebo

ANTHRASIL (Lot No. 10602912) used in these studies was a purified human IgG product manufactured using plasma collected from healthy donors vaccinated with BioThrax^®^ (Anthrax Vaccine Adsorbed) in a 5% solution (59 mg/mL of total protein (>99% is human IgG)) and a potency of 2.73 U/mL by Toxin Neutralization Assay (TNA) based on an anti-AVA reference serum standard obtained from the CDC. Placebo consisted of normal human immune globulin (IGIV) in a 5% solution with 55 mg/mL of total protein manufactured using plasma from normal individuals. Both ANTHRASIL and placebo were manufactured using similar processes and supplied by Emergent BioSolutions Inc., Winnipeg, MB, Canada.

ANTHRASIL and placebo were stored at -35°C to -15°C, and prior to use, were thawed and warmed at 37 ± 2°C for 15 to 30 min and immediately prepared for administration by loading into cassettes.

### Dosing procedures

For all animals, a CADD-Legacy PLUS Model 6500 infusion pump and 50 mL cassette (higher ANTHRASIL doses and IGIV control) were used. ANTHRASIL and placebo were administered by slow intravenous infusion (1.5 to 3.0 mL/kg/h).

#### Rabbit treatment

ANTHRASIL and placebo were administered when PA was detected (≥1.5 ng/mL) in blood using a real-time electrochemiluminescence (ECL) immunoassay as the trigger for treatment on an individual basis. ANTHRASIL was given at a single dose of 15 U/kg and the placebo was administered as a single dose of equivalent volume. For both groups, the infusion duration was approximately 2.5 h. The ANTHRASIL dose was selected based on a previous dose-range study (**S1 File**).

#### NHP treatment

Due to the requirements for slow infusion for ANTHRASIL, an ambulatory infusion system (Strategic Applications, Inc., Libertyville, IL, USA) designed for infusion/pharmacokinetic (PK) studies was used. NHPs were individually housed in cages and remained ambulatory during infusion except when placed in a chair intermittently for short periods to change infusion flow rates. The patency of catheter lines and VAPs was maintained by flushing them with heparinized saline. A sterile infusion line was attached to the infusion pump and attached at the other end to a needle inserted into the VAP. As in the rabbit study, toxemia was used as the trigger for treatment on an individual basis. ANTHRASIL was administered at one of three dose levels (7.5, 15 or 30 U/kg), and the placebo was administered at a volume matching that of the 30 U/kg ANTHRASIL group. The infusion durations were approximately 2, 3 and 4.5 and 4.5 hours for the low, mid and high dose ANTHRASIL and IGIV groups, respectively. The ANTHRASIL doses were based on the rabbit dose-time range study, where the same doses were observed to confer significant protection (**S1 File**).

### Randomization and blinding

Each study was conducted in several cohorts, and animals were randomized to different treatment groups, challenge days (cohorts) and order of challenge (same as cage order) in each cohort. Randomizations were performed with Stata^®^ or with SAS^®^ statistical software using the Plan Procedure.

Both studies were conducted in a blinded fashion such that the personnel involved in the study (study director, technicians performing the dosing, technicians observing the animals, and microbiologists) did not know the treatment group identity of animals during the in-life phase of the study. The study pathologist was blinded until all histopathology slides were read.

### Euthanasia

#### Rabbits

Pre-established criteria for euthanasia of rabbits included the presence of any seizure, severe respiratory distress, and unresponsiveness to touch or external stimuli (moribund). If a drop in hematocrit below 20% was observed, affected rabbits were closely monitored and evaluated by a veterinarian. For euthanasia, rabbits were first sedated with acepromazine (1–6 mg/kg) and then given an overdose of an agent containing pentobarbital.

#### Nonhuman primates

Pre-established criteria for euthanasia of NHP included the presence of any seizure denoting primary central nervous system disease, respiratory distress, dyspnea or forced abdominal respirations, unresponsive to touch or external stimuli, recumbence and weakness, progressive state of depression, > 20% body weight loss, body temperature below 95°F (indicative of shock) or total anorexia with duration longer than 48 hours. Euthanasia was accomplished by first anesthetizing with Telazol^®^ (1–6 mg/kg) intramuscularly, followed by an overdose of a barbiturate administered intravenously or intracardiac injection.

### Clinical observations

#### Rabbits

Prior to the challenge, animals were observed twice daily for clinical signs of illness or disease, including but not limited to reduced food consumption, lethargy, respiratory distress, and seizure activity. Following challenge, rabbits were observed approximately every 6 h for 7 days and thereafter twice daily until the end of the study at Day 36 post-challenge.

#### Nonhuman primates

Clinical signs were observed every 6 h from Days 0 to 10 post-challenge. On Days 11 through 28, clinical observations were recorded twice daily during normal business hours. Following the 28-day study period, surviving animals were transferred to the ABSL-2 facility. An additional twice-daily observation period of 60 days commenced for a total of 88 days of observations post-challenge. Monitoring for adverse reactions was done continuously during infusion of IGIV or ANTHRASIL and any adverse reactions were recorded.

### Body temperature

Body temperature was not measured in the rabbit therapeutic study.

For NHP, body temperature and activity levels were monitored beginning 7–10 days prior to challenge until the end of the in-life phase (Day 28) via AT10TA-D70 DSI telemetry implant.

### Blood collection

#### Rabbits

Blood samples were preferentially taken from the VAP through Day 3 post-challenge. Scheduled blood draws from Day 4 through the end of the study were taken from the ear artery or vein. Prior to blood collection from the ear, rabbits were sedated with acepromazine (1–6 mg/kg), and wintergreen oil was applied to the ear. During terminal collections, blood was collected from the VAP or via cardiac puncture. Whole blood was collected into EDTA tubes for bacteremia and in serum separator tubes (SST) for toxemia by PA detection at various times post-challenge (**Table 1**).

**Table 1:** Schedule of blood collection in rabbit therapeutic efficacy study.

Group	Baseline	Post challenge (PoC)	Prior to Infusion (PrTI)	Post infusion (PoI)
Untreated control	Day -7	Every 6 h during 18–48 h and Days 3, 5, 8, 10. 14, 21 & 28	N/A	N/A
IGIV or ANTHRASIL-treated	Day -3	Every 6 h during 24–48 h and Day 36	Yes	Hours 1,12, 24, and 48, and Days 3, 5, 7, 10, 14, 21, and 28

#### Nonhuman primates

Blood was collected from the femoral, saphenous and cephalic veins. Whole blood was collected in EDTA and SST tubes from all animals from all groups prior to challenge (baseline, Day -7), six-hour intervals from 24 to 72 h and on Days 7, 10, 14, 21 and 28 post-challenge.

### Protective antigen (PA) analysis

Following separation, 80 μL of serum was aliquoted into one vial for toxin analysis by electrochemiluminescence (ECL) immunoassay. Vials were pre-loaded with 10 μL of Protease Inhibitor Cocktail Set III from Calbiochem (Catalogue #539134) and stored at-20°C. Just prior to use, the vials were thawed and 80 μL of serum and 10 μL of 0.2M EDTA (Fisher Catalogue #S311-100) were added sequentially to the vial. Vials were then closed and specimens vortexed for 5 seconds. Specimens were tested using a real-time ECL assay (up to treatment) or were stored at ≤-60°C until assays were performed (post-treatment/ infusion). All frozen, stored specimens were batch-tested.

For both rabbits and NHPs serum specimens were analyzed in real-time according to the collection schedule to identify the onset of toxemia for initiation of treatment. Specimens from the untreated control animals were also tested in real-time if corresponding specimens from the treatment groups were available at that time point. Prior to infusion (PrTI) and post-infusion (PoI) specimens from the treatment groups and specimens from the untreated control group that did not coincide with specimens being analyzed in real-time were preserved frozen and batch-tested at a later time.

### Bacteremia culture

For qualitative bacteremia cultures, 30 to 40 μL of whole blood was plated over blood agar plates and incubated at 37 ± 2°C to determine the presence or absence of colonies consistent with *B*. *anthracis* morphology.

### Quantitative bacteremia by polymerase chain reaction (PCR)

DNA was isolated from 100 μl of every whole blood sample collected and quantitative real-time PCR was performed using primers against a chromosomal target, *rpoB*.

### Complete and differential blood counts

For hematology measurements, blood collected in EDTA tubes was analyzed for total and differential leukocytes with the Advia® 120 and also included the neutrophil: lymphocyte (N/L) ratio.

### Tissue bacterial culture

In rabbits, the presence or absence of *B*. *anthracis* in tissue was assessed as part of histological examinations but not by tissue bacterial culture.

In NHPs, mediastinal lymph nodes, spleen and lung tissue collected at necropsy were homogenized, serially diluted and plated on blood agar.

### Necropsy and histopathology

#### Rabbits

Complete gross necropsies, including the brain were performed on all non-surviving rabbits as well as up to ten randomly selected animals per treatment group that survived to the end of the 36-day post-challenge monitoring period.

#### Nonhuman primates

Gross necropsy and histopathology was conducted on all NHPs with the objective of identifying anthrax—specific lesions and confirmation that deaths in non-survivors were due to *B*. *anthracis* infection. Histopathology was completed on the following organs: mediastinal lymph nodes, spleen, lung, liver, brain/meninges, adrenal glands, kidney and gross lesions observed at necropsy. Sections of sampled tissues from all necropsied animals were processed to slides, stained with hematoxylin and eosin, and examined by a board-certified veterinary pathologist.

### Pharmacokinetic analysis

Serum samples from post-infusion blood collection in rabbits and post-challenge collection in NHPs were assayed by TNA and PA-ELISA. The TNA and ELISA data versus time profiles were evaluated using the non-compartmental analysis module in the WinNonlin software program (Version 6.2, Pharsight Corporation, Mountain View, CA).

## Results

### Efficacy study in rabbits

#### Aerosol challenge

The average aerosol spore exposure dose for all the 110 animals was 194 ± 33 LD_50_ of *B*. *anthracis* (Ames strain) or 97% of the target exposure of 200 LD_50_. The average mass median aerodynamic diameter (MMAD) for all challenge days was 1.20 μM, consistent with lower respiratory tract deposition.

#### ANTHRASIL dosing

The target dose was 15 U/kg. The actual dose delivered to each animal was determined by weighing the infusion syringes or cassettes. All animals received 88% to 114% of the target dose or volume, with the exception of one placebo-treated animal, which was administered 62.4% of the target volume.

#### Survival

**Fig 1:**
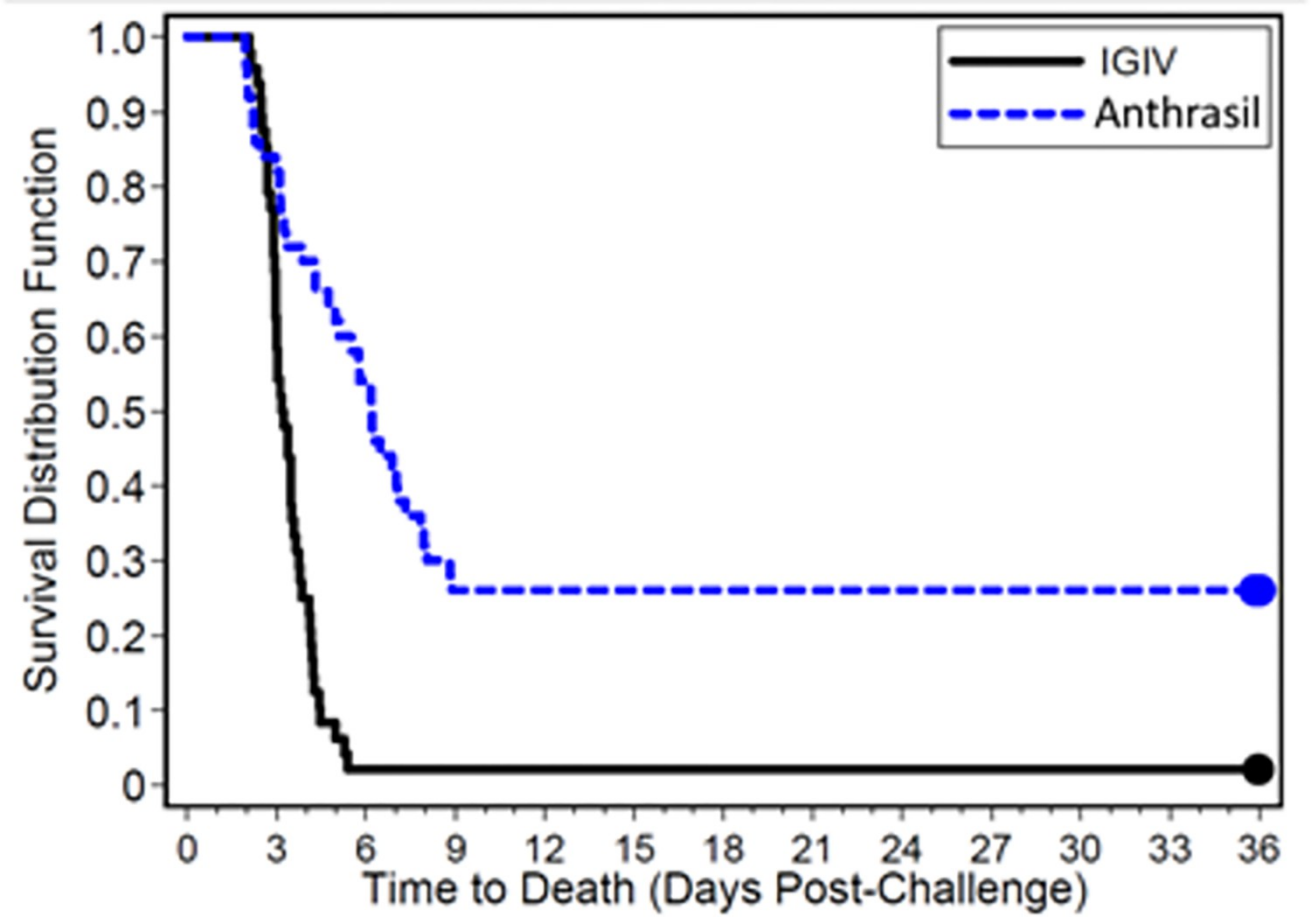
Kaplan-Meier curves representing time-to-death and survival data in *B*. *anthracis*-infected rabbits treated with IGIV or ANTHRASIL. *Bacillus anthracis* spore- challenged rabbits were treated with 15 U/kg ANTHRASIL or a matching volume of IGIV upon detection of toxemia. Only animals that received a full dose of ANTHRASIL or IGIV and were bacteremic at least once prior to treatment were included (Modified intent to treat analysis set).

Two IGIV-treated animals which succumbed to infection did not meet the criteria for inclusion in the MITT set of animals and were excluded from survival analysis. ANTHRASIL significantly enhanced survival (p = 0.0009, Fisher’s exact test) in the MITT set of animals group (26%, 13 of 50) compared to the IGIV group (2%, 1 of 48); ANTHRASIL also increased time to death compared to the IGIV group (p < 0.0001, log-rank test) (**Table 2**, **Fig 1**). ITT data was presented in **S2 File**.

**Table 2:** Proportion of survivors and median time to death in *B*. *anthracis*-infected rabbits treated with IGIV or ANTHRASIL.

Group	Number Survived/N	Proportion of Survivors (95% Confidence Interval)	Fisher’s Exact Test P-value	Median Time to Death in Hours Post-Challenge (95% Confidence Interval)	Log-Rank Test P-value
IGIV	1/48	0.02 (0.00, 0.11)	0.0009*	75.8 (70.7, 83.8)	<0.0001*
ANTHRASIL	13/50	0.26 (0.15, 0.40)		148.5 (113.8, 175.8)	

* = Significant at the 0.05 level; Median time to death was based on Kaplan Meier curve

Only animals that received a full dose of ANTHRASIL or placebo and were bacteremic at least once prior to treatment were included for analysis (Modified Intent-To-Treat (MITT))

A logistic regression model was fitted to the survival data with effects for the treatment group and the toxin level prior to infusion. Based on this model, for animals having the same toxin levels prior to infusion, the odds of survival in the ANTHRASIL group were estimated to be approximately 57 times those in the IGIV group.

#### Clinical observations

Following challenge, the most common clinical signs were lethargy and respiratory abnormalities. The lone survivor from the IGIV treatment group presented with rapid respiration during the first observation on Day 4 and with reduced food consumption on Days 8 and 9. The animal had normal clinical signs for the rest of the study days. Most of the surviving rabbits in the ANTHRASIL-treated group presented with respiratory abnormalities, which were resolved by Day 12. Three animals (one IGIV-treated and two ANTHRASIL-treated) were observed to have labored breathing, sneezing and salivation. These clinical signs were most probably the result of the *B*. *anthracis* infection, stress from handling and the infusion process. None of the three animals with abnormal observations during infusion survived to study completion and all exhibited pathological findings consistent with inhalational anthrax. Body weights remained relatively constant during the study.

#### Toxemia

Rabbits became toxemic as early as 20 h post-challenge (first blood collection after exposure), and all rabbits were toxemic prior to treatment. Median time to positive toxemia was 24.1 h, and 24.3 h for the IGIV and ANTHRASIL-treated groups, respectively. The average time from challenge to toxemia was 26 h for the treated animals and the average time to treatment was 32.4 h post-challenge. The lag time between the onset of toxemia and treatment was due to the time needed to run the ECL assay.

The proportion of toxemic animals (those with ECL results > limit of detection (LOD) (1.0 ng/mL)) after treatment in the IGIV group was significantly greater than that in the ANTHRASIL group (p < 0.05, two-sided Fisher’s Exact test) (**Table 3**). Although PA was not detected in any ANTHRASIL-treated rabbit at 1 h post-infusion (PI), 29 animals had a recurrence of toxemia, particularly from 3 days to 7 days PoI, with 25 succumbing to the disease.

**Table 3:** Proportion of *B*. *anthracis*-infected rabbits toxemic post-treatment with IGIV or ANTHRASIL.

	PTT^a^	1h PoI*	12 h PoI*	24 h PoI*	48 h PoI*	3 D PoI*	5 D PoI	7 D PoI	10 D PoI	14 D PoI	21 D PoI	28 D PoI	36 D PoC	Term*
IGIV	48/48 (100%)	48/48 (100%)	48/48 (100%)	47/47 (100%)	20/20 (100%)	11/11 (100%)	1/1 (100%)	0/1 (0%)	0/1 (0%)	0/1 (0%)	0/1 (0%)	0/1 (0%)	0/1 (0%)	43/43 (100%)
ANTHRASIL	50/50 (100%)	0/50 (0%)	3/50 (6%)	3/44 (7%)	3/38 (8%)	8/35 (23%)	17/27 (63%)	5/16 (31%)	0/13 (0%)	0/13 (0%)	0/13 (0%)	0/13 (0%)	2/13 (15%)	30/35 (86%)

a = Any time point Prior to Treatment (PTT); D = Day; PoC = Post-challenge; PoI = Post-infusion; Term = Terminal

* = Statistically significant difference between groups (p < 0.05, two-sided Fisher’s test)

Only animals that received a full dose of ANTHRASIL or placebo and were bacteremic at least once prior to treatment were included for analysis (Modified Intent-To-Treat (MITT))

**Fig 2:**
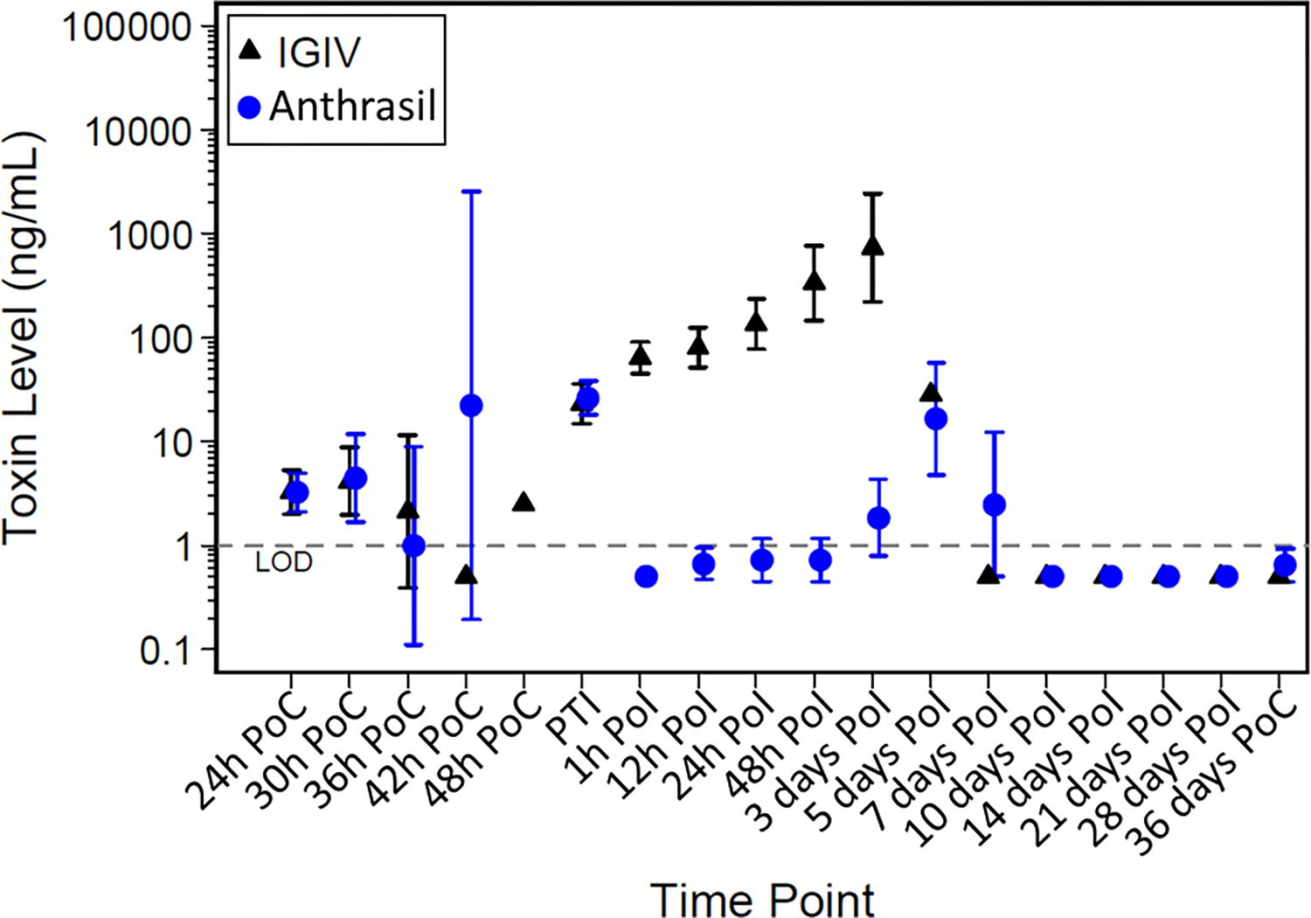
Mean toxin levels in *B*. *anthracis*-infected rabbits treated with IGIV or ANTHRASIL. Plot of geometric means of toxin levels and 95% confidence intervals over time on the log-scale in *B*. *anthracis* spore-challenged rabbits that received a full dose of IGIV or ANTHRASIL and were bacteremic at least once prior to treatment (MITT analysis set). Animals received treatment following the detection of toxemia, which was observed from 24 h to 48 h post-challenge.

At 1 h post-infusion, all toxin levels in the ANTHRASIL group were less than LOD, and the geometric mean toxin level in the IGIV group was significantly greater than LOD (p < 0.0001). From 12 h PoI through 3 days PoI and at the terminal measurement, geometric mean toxin levels in the IGIV group were significantly greater than those in the ANTHRASIL group (p < 0.0001) (**Fig 2**).

Two percent (1 of 48) IGIV-treated animals and 34% (17 of 50) ANTHRASIL-treated animals had toxemia resolved, with median times to resolution of positive toxemia of 155.3 h and 234.2 h post-challenge, respectively (p = 0.0006). No resolution of toxemia was observed in two surviving rabbits in the ANTHRASIL treatment group.

#### Bacteremia

Ninety-eight percent (98 of 100) of the rabbits were bacteremic prior to infusion, and two rabbits from the control (IGIV) group were not bacteremic prior to treatment; these two animals were excluded from the analysis. The lone survivor in the control group was positive for bacteremia prior to the treatment. Median time to positive bacteremia, 24.3 h and 24.7 h for IGIV and ANTHRASIL-treated groups, respectively, was not significantly different between the treatment groups.

At 24 h and Day 3 PoI, the proportion of bacteremic animals in the IGIV group (98% and 95%, respectively) was significantly greater (p = 0.0133 for 24 h PoI and 0.0004 for 3 days PoI) than that in the ANTHRASIL group (82% and 20% at those time points) (**Table 4**). Resolution of bacteremia was significantly better by Day 3 post-infusion in ANTHRASIL-treated animals than IGIV controls (26% vs 2%, p = 0.0074).

**Table 4:** Proportion of *B*. *anthracis*-infected rabbits bacteremic post-treatment with IGIV or ANTHRASIL.

	PT^a^	1h PoI	12 h PoI	24 h PoI*	48 h PoI	3 D PoI*	5 D PoI	7 D PoI	10 D PoI	14 D PoI	21 D PoI	28 D PoI	36 D PoC	Term
**IGIV**	48/48 (100%)	42/48 (98%)	41/48 (88%)	46/47 (98%)	19/20 (95%)	9/11 (95%)	0/1 (0%)	0/1 (0%)	0/1 (0%)	0/1 (0%)	0/1 (0%)	0/1 (0%)	0/1 (0%)	44/47 (94%)
**ANTHRASIL**	50/50 (100%)	36/50 (72%)	41/50 (82%)	36/44 (82%)	29/38 (76%)	7/35 (20%)	13/27 (48%)	3/16 (19%)	0/13 (0%)	0/13 (0%)	0/13 (0%)	0/13 (0%)	0/13 (0%)	37/37 (100%)

a = Any time point prior to treatment; D = Day; PoC = Post-challenge; PoI = Post-infusion; Term = Terminal

* = Statistically significant difference between groups (p < 0.05, two-sided Fisher’s test)

#### Hematology

The hematological examination was performed prior to infusion, on Days 2 and 10 PoI, and on terminal specimens. Mean levels of red blood cells (RBCs), hemoglobin (HGB) and hematocrit (HCT) dropped below normal ranges (~40% reduction over the baseline value) after infusion of ANTHRASIL or IGIV, suggesting the animals were becoming anemic; however, this never became severe enough to warrant euthanasia or other intervention.

While all terminal specimens were below normal ranges, it was not determined if the levels in surviving animals returned to normal as no specimens were collected after Day 10 PoI. The mean levels of RBCs, HGB, and HCT were slightly below normal ranges in ANTHRASIL-treated animals in non-terminal specimens. Post-challenge, white blood cell counts were decreased prior to treatment, while neutrophil counts increased as the study progressed. Lymphocyte counts decreased from baseline prior to treatment but seemed to rebound by 48 h post-infusion. Hematology data from the rabbit study are presented in **S2 File**.

#### Pathology

Gross lesions typical of inhalational anthrax were observed in non-surviving rabbits [33] such as discoloration or foci in the adrenal glands, brain, and large intestines, enlargement of mediastinal lymph nodes, and fluid (effusion) in the thoracic cavity. These findings correlated histologically with hemorrhage, edema, and acute inflammation. No anthrax-related lesions were present in any animal surviving to study completion on Day 36.

Microscopic findings considered consistent with anthrax were also present in all non-surviving animals examined, including acute inflammation, necrosis, hemorrhage, and vascular thrombosis. Large rod-shaped bacteria consistent with *B*. *anthracis* were present in all animals in one or more organs, including the brain, large intestine, lung, mediastinal lymph nodes, and/or spleen. Findings in the brain tissue of non-surviving animals treated with IGIV or ANTHRASIL included the presence of bacteria, hemorrhage, heterophilic meningitis, meningeal vascular necrosis and parenchymal necrosis. Central neuronal necrosis was also observed in some of the rabbits treated with ANTHRASIL but not in the IGIV group (Table 5). There were no microscopic findings in brain tissue collected from the one IGIV-treated survivor or the ANTHRASIL-treated survivors.

**Table 5:** Incidence of brain lesions in IGIV or ANTHRASIL-treated rabbits that died on the study.

Parameter	Untreated control (N = 10)	IGIV-treated (N = 49)	ANTHRASIL-treated (N = 37)
Bacteria	10 (100%)	47 (96%)	29 (78%)
Hemorrhage	0 (0%)	5 (10%)	8 (22%)
Heterophilic meningitis	0 (0%)	3 (6%)	8 (22%)
Meningeal vascular necrosis	1 (10%)	3 (6%)	6 (16%)
Neuronal necrosis	0 (0%)	0 (0%)	5 (14%)
Parenchyma necrosis	1 (10%)	2 (4%)	6 (16%)

#### Pharmacokinetic data

Pharmacokinetics of ANTHRASIL were evaluated in the rabbit study, and systemic exposure with IV infusion of 15 U/kg was confirmed. Twelve of the 16 animals that survived past Day 7 exhibited a humoral immune response to PA, with large increases in concentrations of anti-PA antibodies observed using both the Toxin Neutralization Assay (TNA) and Enzyme-Linked Immune-Sorbent assay (ELISA) (**S3 File**).

### Therapeutic efficacy in cynomolgus macaques

#### Aerosol challenge

The average aerosol exposure dose for all 64 animals was 154 ± 40 LD_50_ of *B*. *anthracis* (Ames strain), or 77% of the target exposure of 200 LD_50_. The MMAD for each of the four challenge days were 0.98 μm, 1.00 μm, 1.11 μm, and 1.09 μm, consistent with lower respiratory tract deposition.

#### ANTHRASIL dosing

The actual dose delivered to each animal was determined by weighing the infusion cassettes. All animals received 95% to 105% of the target dose or volume.

#### Survival

Significantly higher survival rates were observed in animals treated with 15 U/kg or 30 U/kg ANTHRASIL compared to controls (p = 0.0373 and p = 0.0009, respectively) (**Table 6**). Observed survival rates were 6% (1 of 16) in the placebo control and 27% (4 of 15), 44% (7 of 16) and 71% (10 of 14) in the 7.5 U/kg, 15 U/kg and 30 U/kg ANTHRASIL dose groups, respectively. One 7.5 U/kg dose group animal was found unresponsive and was euthanized 24 days following challenge. This animal was negative for bacteremia at all time points, including the terminal one. Further, no gross or histologic lesions typical of anthrax were observed. Based on the lack of findings consistent with *B*. *anthracis* infection, it was concluded that the death in this animal was not caused by anthrax, and therefore, this animal was excluded from survival analysis. Two additional animals treated with 30 U/kg ANTHRASIL were erroneously considered toxemic prior to treatment due to analytical error but were retrospectively determined as not toxemic and excluded from the survival analysis.

**Table 6:** Survival rates of *B*. *anthracis*-infected nonhuman primates treated with IGIV and ANTHRASIL (Intent-to-treat group).

Treatment	No. Survived / Total	Survival Rate (95% Confidence Intervals)	One-Sided Fisher’s Exact Test P-values					
			Unadjusted			Bonferroni-Holm Adjusted		
			ANTHRASIL doses (U/kg)			ANTHRASIL doses (U/kg)		
			7.5	15	30	7.5	15	30
IGIV	1/16	0.06 (0.00, 0.30)	0.1462	0.0186*	0.0003*	0.1462	0.0373*	0.0009*
ANTHRASIL- 7.5 U/kg	4/15^**a**^	0.27 (0.08, 0.55)		0.2692	0.0199*			
ANTHRASIL- 15 U/kg	7/16	0.44 (0.20, 0.70)			0.1235			
ANTHRASIL- 30 U/kg	10/14^**b**^	0.71 (0.42, 0.92)						

* Significant at the 0.05 level; Intent-to-treat (ITT) animals include those that were confirmed toxemic prior to treatment-

^**a**^ one animal that was euthanized due to non-anthrax related cause was excluded

^**b**^ two animals retrospectively found not to be toxemic were excluded

**Fig 3:**
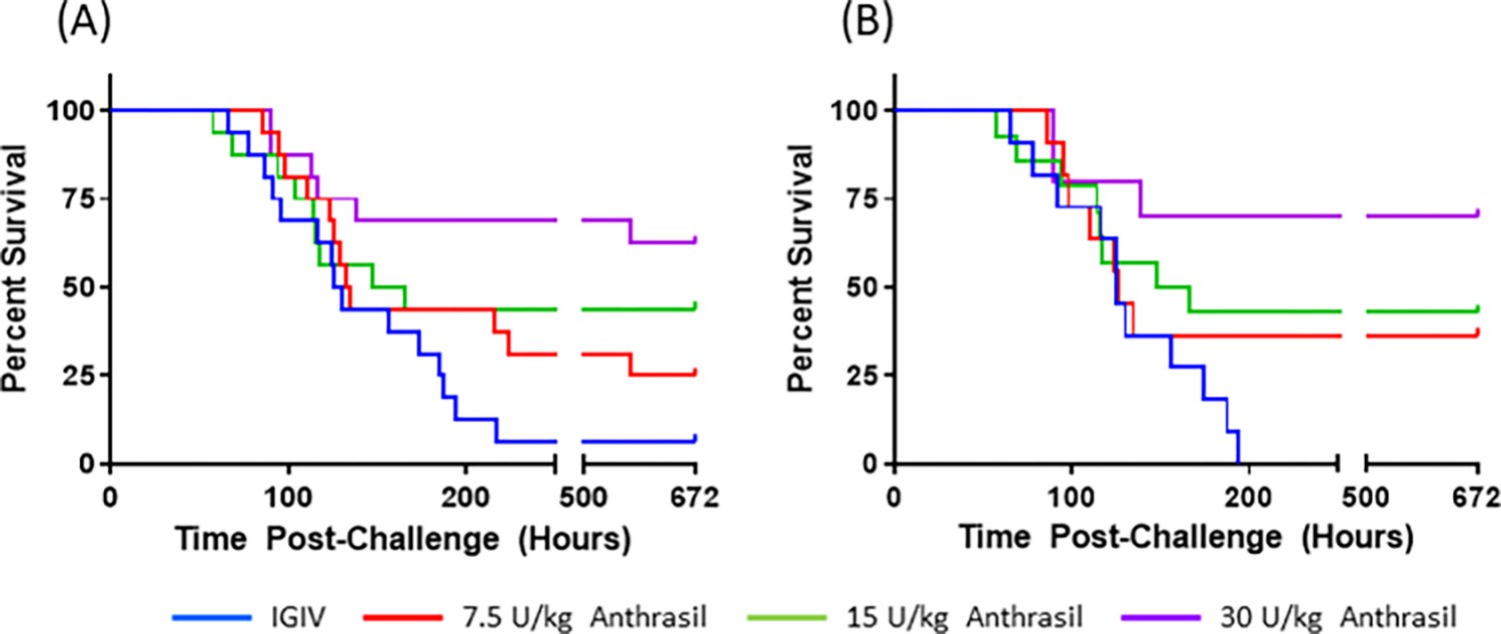
Kaplan-Meier curves representing time-to-death and survival data in *B*. *anthracis*-infected nonhuman primates treated with IGIV or ANTHRASIL. Kaplan-Meier curves for survival and time to death in nonhuman primates. *Bacillus anthracis* spore- challenged cynomolgus macaques were treated with IGIV or three different doses of ANTHRASIL upon detection of toxemia. A) Intent to treat group- all animals that received the treatment B) Modified intent to treat- only animals that were bacteremic at least once prior to treatment were included.

Additionally, using the SAS`S’ LIFETEST procedure, Kaplan-Meier curves were plotted for survival and median times to death were calculated for each group (**Fig 3**).

Analysis of survival rates in the context of toxemia suggests that lower survival rates at lower ANTHRASIL doses correspond to the recurrence of toxemia and bacteremia after initial resolution. Despite a clear decrease in the incidence of toxemia recurrence with increasing doses of ANTHRASIL, no definitive conclusion regarding toxemia recurrence and its relationship with mortality could be made because the majority of animals succumbed from 72 h to Day 7 PoC when there was no toxemia analysis. Although all animals were toxemic prior to treatment, some animals were not bacteremic prior to treatment. Therefore, survival was separately assessed in animals that were bacteremic prior to treatment. Survival rates in this subset of animals were 0%, 36%, 43% and 70% in the Placebo, 7.5 U/kg, 15 U/kg, and 30 U/kg-ANTHRASIL-treated groups, respectively. These survival rates were significantly greater in the 7.5 U/kg, 15 U/kg, and 30 U/kg-ANTHRASIL-treated groups compared to controls (p ≤ 0.0451) (**Table 7**).

**Table 7:** Survival rates of *B*. *anthracis*-infected nonhuman primates treated with IGIV and ANTHRASIL (Modified intent-to-treat group).

Treatment	No. Survived/ Total	Survival Rate (95% Confidence Intervals)	One-Sided Fisher’s Exact Test P-values					
			Unadjusted			Bonferroni-Holm Adjusted		
			ANTHRASIL doses (U/kg)			ANTHRASIL doses (U/kg)		
			7.5	15	30	7.5	15	30
Placebo	0/11	0.00 (0.00, 0.28)	0.0451*	0.0170*	0.0010*	0.0451*	0.0339*	0.0031*
ANTHRASIL—7.5 U/kg	4/11	0.36 (0.11, 0.69)		0.5340	0.1349			
ANTHRASIL—15 U/kg	6/14	0.43 (0.18, 0.71)			0.1846			
ANTHRASIL—30 U/kg	7/10	0.70 (0.35, 0.93)						

* Significant at the 0.05 level

Modified Intent-to-treat (MITT) animals excluded those that were not bacteremic prior to treatment- additionally, one animal that was euthanized due to non-anthrax related clinical condition and two animals retrospectively found not to be toxemic were excluded

#### Body temperature and activity

Animals were considered to have a significant increase in body temperature (SIBT) when six consecutive measurements were above animal baseline body temperature (2 standard deviations of baseline adjusted temperatures over the average baseline adjusted body temperature). Recovery from elevated temperature (return to baseline temperature) is defined as the observation of 6 consecutive temperature measurements below the SIBT.

A minority of animals (38%) in each of the placebo, 7.5 U/kg and 15 U/kg ANTHRASIL-treated groups and 25% in the 30 U/kg ANTHRASIL-treated group exhibited SIBT before treatment. Neither the proportion of animals exhibiting SIBT prior to treatment nor the time between treatment initiation and return of temperature consistent with baseline was significantly different between groups.

#### Clinical observations

NHPs exhibited clinical signs of infection, including lethargy and hunched posture, not eating, stool abnormalities, and respiratory abnormalities following infection. All (100%) animals exhibited inappetence within a week following the challenge, and 94% of animals in each treatment group exhibited stool abnormalities within the first eight days following the challenge. High proportions of animals in all groups (88% of IGIV-treated, 94% of 7.5 U/kg ANTHRASIL-treated, 100% of 15 U/kg ANTHRASIL-treated and 94% 30 U/kg ANTHRASIL-treated) had activity abnormalities. Relatively few animals exhibited respiratory abnormalities (44% of IGIV-treated, 19% of 7.5 U/kg ANTHRASIL-treated, 25% of 15 U/kg ANTHRASIL-treated and 19% of 30 U/kg ANTHRASIL-treated animals). The abnormalities were persistent in non-survivors. In contrast, only sporadic observations of inappetence and minor stool abnormalities were noted in the surviving animals a week after infection.

#### Body weights

Over the first week post-*B*. *anthracis spore* exposure, most surviving animals exhibited slight decreases (~ 8% across all groups) in body weight, after which no remarkable changes in body weights were observed.

#### Toxemia

Treatment was initiated individually when an animal exhibited a PA assay result greater than the lower limit of quantification (1.5 ng/mL). Consequently, all animals exhibited PA in circulation before treatment when the results were analyzed. However, retrospectively it was determined that two animals treated with 30 U/kg ANTHRASIL were not toxemic prior to treatment and were therefore excluded from the survival analysis. At the time of treatment, circulating PA concentrations in various groups ranged from 1.61 ng/mL to 18.20 ng/mL (IGIV-treated), 1.90 ng/mL to 30.85 ng/mL (7.5 U/kg ANTHRASIL-treated),2.28 ng/mL to 150.33 ng/mL (15 U/kg ANTHRASIL-treated) or 1.92 ng/mL to 21.55 ng/mL (30 U/kg ANTHRASIL™-treated). Circulating PA levels were reduced to undetectable levels (< LOD) by ANTHRASIL in all but two animals (one each from the 7.5 U/kg and 15 U/kg ANTHRASIL-treated groups) when the first serum sample was assessed following treatment.

**Fig 4:**
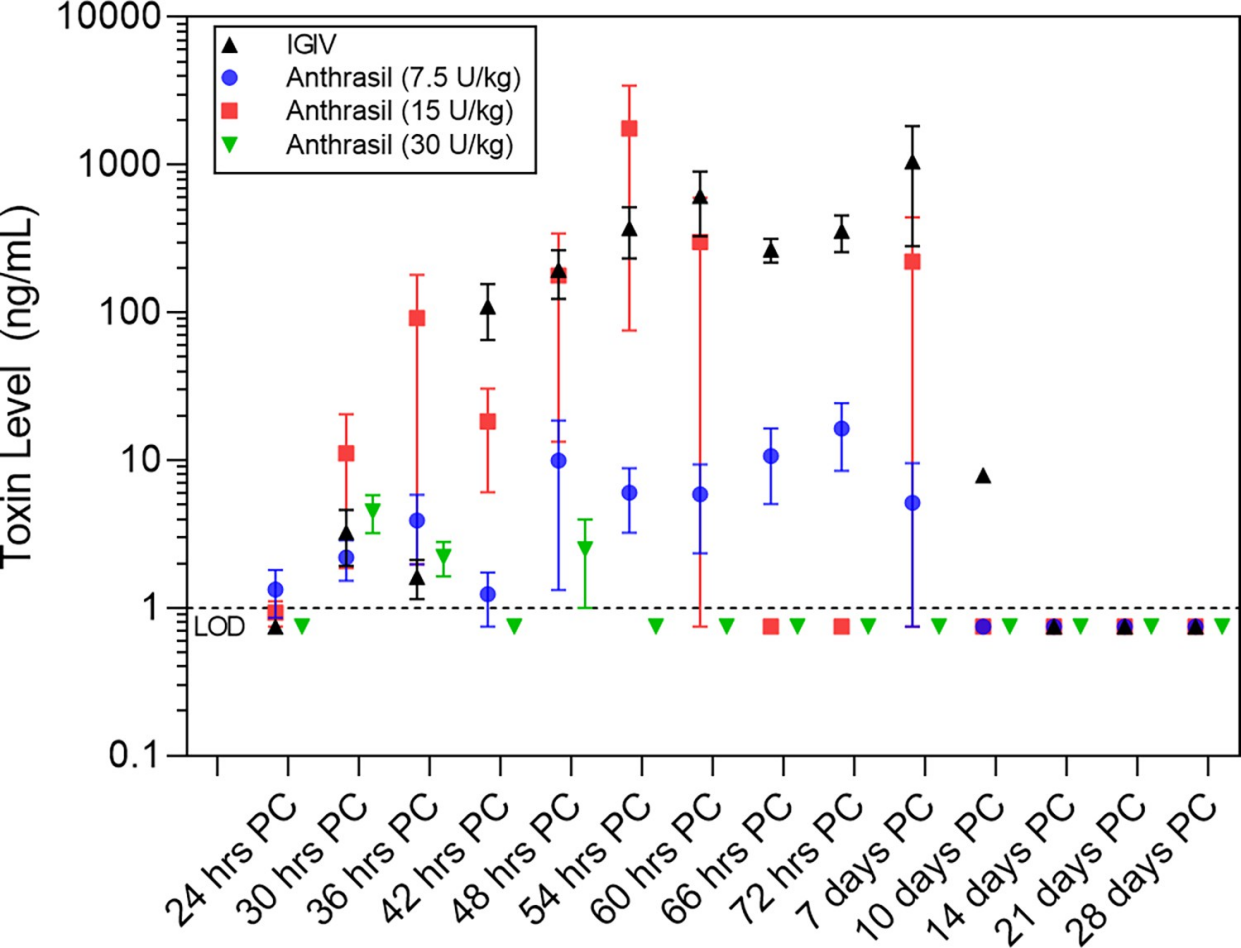
Mean toxin levels in *B*. *anthracis*-infected nonhuman primates treated with IGIV or three different doses of ANTHRASIL. Plot of geometric means of toxin levels and 95% confidence intervals over time on the log-scale in *B*. *anthracis* spore-challenged NHPs that received a full dose of IGIV or one of three doses of ANTHRASIL and were bacteremic at least once prior to treatment (Modified-intent-to-treat analysis set). Animals were treated following the detection of toxemia, which was observed from 24 h to 54 h post-challenge, and infusions began within 6 h (approximately 30 h to 60 h post-challenge).

The proportion of toxemic animals post-treatment was significantly greater in IGIV control group than in any other treatment group from 6 to 24 and from 24 to 36 hours post-treatment (p < 0.0001, p = 0.0065, respectively). Additionally, there was a discernible dose-dependent decrease in the proportion of toxemic animals from 6 h PoI onwards. A recurrence of toxemia was observed in some animals that received 7.5 and 15 U/kg ANTHRASIL (8 of 15 and 2 of 15 animals, respectively) (**Fig 4**).

#### Bacteremia (blood culture)

Before treatment, 69% of the IGIV group, 69% of the 7.5 U/kg dose group, 88% of the 15 U/kg dose group and 63% of the 30 U/kg dose group were bacteremic. On Day 24, one non-surviving animal in the 7.5 U/kg dose group exhibited complications with the telemetry implant, with no attribution to anthrax based on pathology results. Two animals in the 30 U/kg dose group were not bacteremic by blood culture at treatment and were excluded from survival analysis.

IGIV-treated animals had significantly higher bacteremia rates than animals treated with 30 U/kg ANTHRASIL at all time points tested (p ≤ 0.0101). IGIV-treated groups also had higher bacteremia rates from 12 to 30 hours post-treatment and from 12 to 24 hours post-treatment than the groups treated with the 15 U/kg (p ≤ 0.0304) and 7.5 U/kg of ANTHRASIL (p = 0.0242), respectively.

In contrast to toxemia data, post-treatment recurrence of bacteremia did not differ remarkably between the three dose levels of ANTHRASIL. Approximately 50% of animals treated with 7.5 or 15 U/kg ANTHRASIL exhibited recurrence of bacteremia compared to 70% of animals treated with 30 U/kg ANTHRASIL, despite enhanced survival with higher ANTHRASIL doses.

#### Complete and differential blood cell counts

Increases in total WBC and neutrophil counts, decreased lymphocyte counts and an increased N/L ratio were observed across all groups post-challenge. The delayed onset, moderate correlation with toxemia/bacteremia onset and lack of significant differences in the proportion of animals across different groups at any time-point post-treatment limited the value of these data as either a trigger for treatment or assessment of disease progression (**S4 File**).

#### Pathology

All gross lesions observed in non-surviving NHPs were typical of inhalation anthrax in cynomolgus macaques [45], correlating microscopically with varying combinations of tissue necrosis, hemorrhage, edema, fibrino-neutrophilic inflammation, and the presence of large rod-shaped bacteria consistent with *B*. *anthracis* in multiple organs. Microscopic findings considered consistent with anthrax were also present in all 40 non-surviving NHPs.

Control animals had a greater incidence and/or severity of parenchymal organ lesions than did ANTHRASIL-treated animals. When present, meningeal lesions in IGIV-treated control animals were largely confined to minimal to mild hemorrhage, with no evidence of inflammatory infiltrate. In contrast, the subset of ANTHRASIL-treated non-surviving animals exhibited a dose-dependent increase in the incidence and severity of inflammatory lesions in the brain/meninges compared to IGIV-treated control animals. In addition, non-surviving ANTHRASIL-treated animals had more numerous and widespread bacteria observed in the brain, while bacteria in the brain of non-surviving IGIV-treated control animals were often confined to blood vessels or the immediate perivascular space. Similar observations of increased incidence and severity of brain lesions (hemorrhage and bacteria) in cynomolgus macaques have been reported in animals treated with monoclonal antibodies against PA [26].

While the survival rates were greater in ANTHRASIL-treated cynomolgus macaques compared to IGIV-treated controls, a high percentage of non-survivors in ANTHRASIL-treated groups, exhibited lesions characterized by a mixed accumulation of neutrophils and fibrin in the meningeal space, typical of an acute immune reaction that developed hours to days after the onset of local bacterial invasion. This appeared to be dose-dependent, with 27% (3 of 11) of 7.5 U/kg dose, 66% (6 of 9) of 15 U/kg dose and 100% (5 of 5) of 30 U/kg dose of non-surviving ANTHRASIL-treated animals exhibiting lesions resulting from neutrophilic or fibrin inflammation. Of the IGIV control animals that succumbed, 53% were documented to have bacteria found in the brain, and 80% exhibited hemorrhage, but none showed inflammatory lesions. The cause of these findings could not be determined.

#### Pharmacokinetic data

Pharmacokinetic analysis from a few NHPs was performed by TNA as non-GLP post-hoc testing outside the scope of the original study protocol. As the blood collection time points in this study were relative to time of *B*. *anthracis* challenge rather than the time of completion of infusion, the data should be interpreted with caution. Nonetheless, PK data in the *B*. *anthracis*-challenged NHPs confirmed the exposure to ANTHRASIL, which increased in a dose-proportionate manner with no gender difference (**S3 File**).

## Discussion

ANTHRASIL is a purified human IgG product manufactured using plasma collected from healthy donors vaccinated with AVA (Anthrax Vaccine Absorbed) and consists of polyclonal neutralizing antibodies against the PA antigen of *B*. *anthracis*. ANTHRASIL has been licensed for the treatment of inhalational anthrax under the “Animal Rule” by the FDA based on the demonstrated efficacy in animal models of inhalational anthrax and safety in animals and humans. The animal model data from rabbits and NHPs described here formed the evidence of efficacy for licensure under this regulatory path.

When given after the onset of systemic disease based on the detection of PA toxin in circulation, ANTHRASIL demonstrated a statistically significant survival benefit compared to placebo control groups. The mean *B*. *anthracis* spore exposure doses in both species were close to the target dose of 200 LD_50_ (194 LD_50_ in rabbits and 154 LD_50_ in NHPs).

The treatment dose of 15 U/kg ANTHRASIL in rabbits was based on a previous dose-time range study in which animals were administered ANTHRASIL prophylactically at fixed time points (20 h or 30 h) post-exposure (**S1 File**).

The therapeutic intervention study in rabbits described here involved challenging animals with a lethal aerosol dose of *B*. *anthracis* spores and initiating individual treatment following real-time detection of the serum PA. Toxemia was used as the trigger for treatment; the validity of this trigger was confirmed by the post-hoc demonstration that 98% of treated rabbits on study were bacteremic prior to treatment. This was also consistent with the previous dose-range study, wherein close to 80% of the animals were bacteremic 30 h post-challenge. Comer et al. [27] used a significant increase in body temperature (SIBT) as a trigger to treat rabbits and reported that PA detection in serum corresponded with positive blood cultures for 68% of the rabbits. In their study, the mean times from challenge to SIBT and toxemia were 27 h and 28 h, respectively. The mean time to toxemia in treated animals in our study, 25.97 h was close to that reported earlier. Additionally, while the times from challenge to detectable levels of PA were relatively consistent, a wide range (e.g., 15.6 to 4,365.5 ng/mL) in the levels of circulating PA was observed. It should be noted that the data from the untreated group were not used in the statistical evaluation of the efficacy of ANTHRASIL, but instead confirmed the model characterization data such as 100% mortality with the challenge dose used, time to death and onset of various parameters (**S2 File**).

This study was randomized and blinded as per Animal Rule guidance and demonstrated that ANTHRASIL-treatment significantly enhances survival compared to IGIV controls in a therapeutic setting. Treatment with ANTHRASIL significantly decreased levels of circulating PA, and the proportion of rabbits found to be toxemic post-infusion (p < 0.0001), consistent with the mechanism of action of polyclonal anti-sera directed against PA. Interestingly, two ANTHRASIL-treated rabbits that survived were positive for toxemia at the end of the study. As neither of these animals were bacteremic after treatment, it is unclear if the low levels of PA detected (> LOD of 1 ng/mL but < LLOQ of 5 ng/mL) at the end of the study were false positives.

While not as quick as antimicrobials that eliminate bacteremia within 24 hours of treatment [20, 47], ANTHRASIL treatment also significantly decreased the proportion of bacteremic animals beginning the third day post-infusion, as well as the time to resolution of bacteremia after infusion. As *B*. *anthracis* toxins are known to inhibit host immune cells, the clearance of bacteria suggests that ANTHRASIL neutralizes the inhibitory effects of the toxins, allowing the host immune response to clear the infection more efficiently [15].

As in the rabbit study, nonhuman primates also were individually treated with ANTHRASIL following the detection of PA in real-time. Since no dose-ranging study was conducted previously in NHPs, three doses of ANTHRASIL were evaluated in this study. Henning et al. [32] also used toxemia as the trigger for treatment with a monoclonal antibody in cynomolgus macaques challenged with 198–950 LD_50_, observing mean times to positive PA-ECL for treated and untreated animals of 37.59 and 38.69 h, respectively. Although the range of challenge dose in our study (98–277 LD_50_) was narrower, we observed a similar time to onset of toxemia. Henning et al. observed a100% correlation between toxemia and bacteremia immediately prior to treatment. However, in our current study, this correlation was 69% for Groups 1 and 2, 88% in Group 3 and only 63% in Group 4, prior to treatment. The reason for this lower correlation is unknown. Nonetheless, both studies confirmed that PA detection is the earliest and the optimal trigger for therapeutic intervention. Observation by Henning et al. that other clinical signs such as increased body temperature, WBC count and lethargy had delayed onset was also confirmed in our study, as indicated by the low proportion of NHPs positive for these parameters prior to the treatment (**S4 File**).

Consistent with observations in rabbits, treatment with ANTHRASIL after the onset of toxemia in nonhuman primates resulted in significantly enhanced survival and reduced levels of toxemia, incidence of bacteremia and clinical signs of infection. This contrasts with monoclonal antibody treatment, in which monoclonals enhanced survival but did not significantly reduce toxemia [32]. It may be noted that despite the lower proportion of animals in the 15 U/kg dose group, which remained toxemic post-treatment and had reduced recurrence of toxemia, the mean observed toxin levels in this group were higher than in the 7.5 U/kg dose group. This was attributed to very high toxin levels in two animals of 15 U/kg group that succumbed to infection. The dose-dependent decrease in toxin recurrence suggests that higher doses of ANTHRASIL more efficiently sustain toxin neutralization than lower doses. These data indicate that ANTHRASIL effectively reduced toxemia immediately after infusion, but higher doses of ANTHRASIL provide more sustained toxin neutralization. In addition to the toxemia, the incidence of bacteremia was also reduced in a dose-dependent manner, indicating that treatment with a high dose of ANTHRASIL may assist in limiting the progression of the disease. However, there was no difference in the rate of recurrence of bacteremia among the three doses of ANTHRASIL. Nonetheless, there was a dose-dependent increase in survival, possibly, due to a more efficient and sustained neutralization of toxin by ANTHRASIL. Animals which did not have post-treatment recurrence of bacteremia invariably survived, emphasizing the importance of completely negative bacteremia (and consequent lack of toxin) as a predictor of survival.

While the gross lesions were similar between rabbits and NHPs, histopathological findings in the brain were different in NHPs, wherein a trend towards the development of meningeal inflammation was observed in the higher dose groups. Meningeal lesions in IGIV-treated control NHPs were largely confined to minimal to mild hemorrhage with no evidence of inflammation. In addition, more numerous and widespread bacteria were observed in the brains of ANTHRASIL-treated NHPs that succumbed to disease, while bacteria in the brains of IGIV-treated control NHPs were often confined to blood vessels or the immediate perivascular space. These findings are consistent with those by Migone [36], O’Shaughnessy and Gopinath [48], who observed a similar increased incidence and severity of brain lesions (hemorrhage and bacteria) in cynomolgus macaques treated with Raxibacumab or obiltoxaximab, two licensed monoclonal antibodies against PA, compared to placebo- treated NHPs that succumbed to the disease. There was no clear correlation between persistent bacteremia and brain lesions. In the lower ANTHRASIL doses, some of the non-surviving animals that were negative post-treatment also had brain lesions. At the highest ANTHRASIL dose, all animals were negative post-treatment, but a higher proportion of animals had brain lesions. It should be noted that all animals that died on the study were positive for bacteremia at the terminal sample and there were no samples from 72 hours to Day 7 post-challenge when these animals succumbed. There was also no correlation between the terminal toxemia levels and incidence and severity of brain lesions (Data not shown).

The lack of similar brain lesions in rabbits has been suggested by Zaucha et al. [45] as attributable to the greater susceptibility of rabbits to *B*. *anthracis* infection, which is associated with reduced leucocytic response to the bacilli and a rapid progression to death, further limiting the development of leucocytic infiltrates in response to the basic lesion of hemorrhage and necrosis. Indeed, in our study, an increased incidence of neuronal necrosis but no inflammation in the brain was observed in rabbits treated with ANTHRASIL but not in the IGIV group. Given the significant survival benefit provided by ANTHRASIL, it is clear that the protective effects of ANTHRASIL treatment outweigh any potential deficiencies in clearing infection from the meningeal compartment in a clinical setting. Further, as ANTHRASIL is used as an adjunct to antimicrobial treatment, the risks of persistent bacteremia and associated brain pathology are minimized.

Exposure to ANTHRASIL was confirmed in a formal PK analysis in rabbits and non-GLP post hoc PK analysis in NHPs (**S3 File**).

Overall, an improvement in survival rates was observed following a single administration of ANTHRASIL after the detection of toxemia compared to placebo in both rabbit and cynomolgus macaque models of inhalational anthrax. The improved survival in both models was associated with an immediate and efficient reduction in toxemia. This is consistent with the use of ANTHRASIL for the treatment of toxemia associated with inhalational anthrax, and modeling by Rubinson [49] suggests that antitoxins like ANTHRASIL can extend the time available to initiate antimicrobial treatment after infection.

One drawback of the NHP study is that it was not powered to detect the significant differences between different doses; however, the goal was to evaluate if a dose of ANTHRASIL that mimics the exposure in humans with the estimated clinical dose would confer significant protection over the control group. Further, determining differences between treatment groups would require a very high number of nonhuman primates which is prohibitive for ethical reasons. Further, since these studies were conducted with a focus on meeting the regulatory requirements for drug approval, no detailed pathophysiological mechanisms were explored for the observed differences in brain lesions between rabbits and NHPs.

It should also be noted that the studies described in this report involved a very high challenge dose and relied on a single dose of ANTHRASIL for efficacy, representing the most stringent model for product evaluation and yet significantly improved survival was observed after treatment with ANTHRASIL. In a clinical scenario, where patients receive a multitude of therapies, even better treatment outcome could be expected.

Based on the work presented here, ANTHRASIL was approved by FDA in 2015 [50, 51] and was the first polyclonal plasma product approved and the second product approved under the new regulatory pathway, the Animal Rule for inhalational anthrax.

## Supporting information

S1 File Rabbit dose-range efficacy study. (PDF)

S2 File Additional data from rabbit therapeutic study. (PDF)

S3 File Pharmacokinetics of ANTHRASIL in the rabbits and NHP. (PDF)

S4 File Additional data from NHP therapeutic study. (PDF)
