# Supplementary material for: Efficacy of ANTHRASIL (Anthrax Immune Globulin Intravenous (Human)) in rabbit and nonhuman primate models of inhalational anthrax: Data supporting approval under animal rule
Source: PLoS One. 2023 Mar 17;18(3):e0283164. doi: 10.1371/journal.pone.0283164 (PMC10022752; doi:10.1371/journal.pone.0283164)
Supplement: S1 File — (PDF) [file pone.0283164.s001.pdf]

Supplementary data: S-1 (Rabbit dose-range efficacy study)

Table S-1A: Survival rate in rabbit dose-time range study

Group	Treatment			# Survived/ Total	Survival Rate (95% Confidence Interval)
	Test Material	Dose (AIG U/kg)	Treatment Time (Hours Post-Challenge)		
1	None	N/A	N/A	0/10	0.00 (0.00, 0.31)
2	IVIG	N/A	20 (+/- 1)	0/14	0.00 (0.00, 0.23)
3	IVIG	N/A	30 (+/- 1)	0/14	0.00 (0.00, 0.23)
4	NP-015	7.5	20 (+/- 1)	8/14	0.57 (0.29, 0.82)
5	NP-015	7.5	30 (+/- 1)	4/14	0.29 (0.08, 0.58)
6	NP-015	15	20 (+/- 1)	11/14	0.79 (0.49, 0.95)
7	NP-015	15	30 (+/- 1)	6/14	0.43 (0.18, 0.71)
8	NP-015	30	20 (+/- 1)	13/14	0.93 (0.66, 1.00)
9	NP-015	30	30 (+/- 1)	5/14	0.36 (0.13, 0.65)

N/A Not available; no treatment received.

Table S-1B: Results of Fisher's Exact Test Comparisons for Each Treatment Group

Group	One-sided Fisher's Exact P-value, Comparison to the Untreated Control Group (Group 1)		One-sided Fisher's Exact P-value, Comparison to the Control Group Treated at 20 Hours Post-Challenge (Group 2)		One-sided Fisher's Exact P-value, Comparison to the Control Group Treated at 30 Hours Post-Challenge (Group 3)	
	Unadjusted	Bonferroni -Holm Adjusted	Unadjusted	Bonferroni-Holm Adjusted	Unadjusted	Bonferroni-Holm Adjusted
4	0.0041*	0.0163**	0.0010*	0.0010**		
5	0.0942	0.0942			0.0489*	0.0489**
6	0.0001*	0.0007**	<0.0001*	<0.0001**		
7	0.0223*	0.0669			0.0080*	0.0239**
8	<0.0001*	<0.0001**	<0.0001*	<0.0001**		
9	0.0471*	0.0942			0.0204*	0.0407**

* Survival rate in the treatment group was significantly greater than the rate in the control group at the 0.05 level of significance.

** Survival rate in the treatment group was significantly greater than the rate in the control group at the Bonferroni-Holm adjusted 0.05 level of significance.

Table S-1C: Incidence of Abnormal Clinical Observations in Rabbits Treated at Fixed Time Points After Aerosol Challenge with *B. anthracis*

Group	Treatment	Hematuria	Lethargy	Inappetence
1	Untreated	0% (0/10)	70% (7/10)	80% (8/10)
2	Placebo- 20h	79% (11/14)	57% (8/14)	57% (8/14)
3	Placebo- 30h	79% (11/14)	71% (10/14)	29% (4/14)
4	Anthrasil- 7.5 U/kg (20 h)	0% (0/14)	29% (4/14)	50% (7/14)
5	Anthrasil- 7.5 U/kg (30 h)	7% (1/14)	64% (9/14)	71% (10/14)
6	Anthrasil- 15 U/kg (20 h)	7% (1/14)	0% (0/14)	50% (7/14)
7	Anthrasil- 15 U/kg (30 h)	14% (2/14)	57% (8/14)	79% (11/14)
8	Anthrasil- 30 U/kg (20 h)	29% (4/14)	21% (3/14)	57% (8/14)
9	Anthrasil- 30 U/kg (30 h)	71% (10/14)	64% (9/14)	71% (10/14)

Table S-1D: Number of Animals Exhibiting SIBT, Positive PA Level, and Positive Bacteremia Culture Prior to Treatment

Group	Number Exhibiting SIBT Prior to Treatment/Total Number of Animals	Number With Positive PA Prior to Treatment/Total Number of Animals	Number Bacteremic Prior to Treatment/Total Number of Animals
2	0/14 (0%)	0/14 (0%)	NT
3	7/14 (50%)	10/14 (71%)	10/14 (71%)
4	0/14 (0%)	0/14 (0%)	NT
5	8/14 (57%)	11/14 (79%)	11/14 (79%)
6	0/14 (0%)	2/14 (14%)	NT
7	6/14 (43%)	10/14 (71%)	9/14 (64%)
8	0/14 (0%)	1/14 (7%)	NT
9	7/14 (50%)	12/14 (86%)	12/14 (86%)

NT – Not tested (first sampling was 24 hours post-challenge)

Survival rates were significantly ($p < 0.05$) greater in rabbits treated intravenously with any of three different doses of ANTHRASIL compared to placebo controls. Enhanced survival was associated with earlier treatment time and the observation of lower toxemia at the time of treatment. In addition to enhanced survival, time-to-death was also extended in ANTHRASIL -treated groups. At 30 h post- anthrax exposure, the majority of the animals were bacteremic and 25% of those animals were protected by ANTHRASIL treatment, demonstrating the therapeutic efficacy of ANTHRASIL. Based on the highest survival following delayed treatment at 30 h post-

challenge and lower incidence of adverse effects such as hematuria and lethargy, the 15 U/kg dose of ANTHRASIL in this dose-time study was considered optimal and was used in the rabbit and NHP efficacy studies described in this manuscript.
