# Supplementary material for: Efficacy of ANTHRASIL (Anthrax Immune Globulin Intravenous (Human)) in rabbit and nonhuman primate models of inhalational anthrax: Data supporting approval under animal rule
Source: PLoS One. 2023 Mar 17;18(3):e0283164. doi: 10.1371/journal.pone.0283164 (PMC10022752; doi:10.1371/journal.pone.0283164)
Supplement: S2 File — (PDF) [file pone.0283164.s002.pdf]

Supplementary data: S-2 (Additional data from rabbit therapeutic study)

Table S-2A: Experimental Design of Rabbit Therapeutic Efficacy study

Group	No. of animals	Treatment	Treatment dose	Treatment time
1	10	None	None	None
2	50	IGIV	Volume equivalent to ANTHRASIL	Onset of toxemia
3	50	ANTHRASIL	15 U/kg	Onset of toxemia

Table S-2B: Descriptive Statistics for Red Blood Cell Count, Hemoglobin, and Hematocrit by Group and Time Point (MITT analysis Set)

Parameter	Group	Time Point	N	Arithmetic Mean	Std. Dev.
Red Blood Cells (10^6 cells/µL) Normal Range: 4.20 – 6.70 x 10^6/µL	IGIV	Baseline	48	5.95	0.86
		PTI	48	5.43	0.78
		48 Hours PI	20	3.98	0.89
		10 Days PI	1	2.57	NA
		Terminal	37	3.02	0.50
	NP-015	Baseline	50	5.95	0.84
		PTI	50	5.39	0.76
		48 Hours PI	38	4.50	0.73
		10 Days PI	13	4.13	1.15
		Terminal	32	3.41	0.60
	Untreated Control	Baseline	10	5.90	1.87
Hemoglobin (HGB, g/dL) Normal Range: 9.5 – 14.5 g/dL	IGIV	Baseline	48	12.5	1.8
		PTI	48	11.5	1.7
		48 Hours PI	20	8.8	2.0
		10 Days PI	1	5.8	NA
		Terminal	37	7.5	1.2
	NP-015	Baseline	50	12.5	1.8
		PTI	50	11.4	1.6
		48 Hours PI	38	9.5	1.5
		10 Days PI	13	8.7	2.4
		Terminal	32	7.9	1.4
	Untreated Control	Baseline	10	12.6	4.0
Hematocrit (HCT, %) Normal Range: 27.2 – 45.9%	IGIV	Baseline	48	36.6	5.2
		PTI	48	33.5	4.7
		48 Hours PI	20	23.7	5.1
		10 Days PI	1	20.7	NA
		Terminal	37	20.8	3.4
	NP-015	Baseline	50	36.7	5.2
		PTI	50	33.1	4.7
		48 Hours PI	38	26.9	4.4
		10 Days PI	13	27.0	7.5
		Terminal	32	23.8	4.2
	Untreated Control	Baseline	10	36.8	11.6

Note: Anthrasil was referred to as NP-015

Table S-2C: Descriptive Statistics for White Blood Cell Count, Neutrophil Count and Lymphocyte Count by Group and Time Point (MITT Analysis Set)

Parameter	Group	Time Point	N	Arithmetic Mean	Std. Dev.
White Blood Cell Count (10^3 cells/µL) Normal Range: 2.90 - 8.10 x 10^3/µL	IGIV	Baseline	48	8.04	1.16
		PTI	48	4.70	0.68
		48 Hours PI	20	10.31	2.31
		10 Days PI	1	9.38	NA
		Terminal	37	13.58	2.23
	NP-015	Baseline	50	7.61	1.08
		PTI	50	5.02	0.71
		48 Hours PI	38	7.85	1.27
		10 Days PI	13	8.56	2.37
		Terminal	32	14.32	2.53
	Untreated Control	Baseline	10	8.32	2.63
Neutrophil Count (10^3 cells/µL) Normal Range: 0.80 - 2.90 x 10^3/µL	IGIV	Baseline	48	1.65	0.24
		PTI	48	2.15	0.31
		48 Hours PI	20	3.39	0.76
		10 Days PI	1	3.08	NA
		Terminal	37	3.87	0.64
	NP-015	Baseline	50	1.52	0.22
		PTI	50	2.21	0.31
		48 Hours PI	38	2.69	0.44
		10 Days PI	13	3.22	0.89
		Terminal	32	4.99	0.88
	Untreated Control	Baseline	10	1.79	0.57
Lymphocyte Count (10^3 cells/µL) Normal Range: 2.20 – 5.30 x 10^3/µL	IGIV	Baseline	48	5.57	0.80
		PTI	48	2.24	0.32
		48 Hours PI	20	6.10	1.36
		10 Days PI	1	5.12	NA
		Terminal	37	7.91	1.30
	NP-015	Baseline	50	5.27	0.74
		PTI	50	2.46	0.35
		48 Hours PI	38	4.15	0.67
		10 Days PI	13	4.18	1.16
		Terminal	32	6.73	1.19
	Untreated Control	Baseline	10	5.80	1.83

Table S-2D: Model characterization data from anthrax-challenged untreated control rabbits

Animal ID	Challenge Dose (LD50)	Time of Positive ECL (hr PC)	PA levels at PTI (ng/mL)	First Positive Bacteremia	Time to death (days)	Terminal Bacteremia	Terminal PA Levels (ng/mL)
L49303	206	36	NA	36	3.86	+	738.75
L49360	157	30	NA	30	5.65	+	6512.50
L49311	205	30	NA	30	3.69	+	9650.50
L49363	205	24	NA	30	2.70	+	4725.33
L49321	195	24	NA	24	2.44	+	412.67
L49375	191	24	NA	24	4.39	+	NS
L43316	221	24	NA	24	2.86	+	NS
L43352	247	24	NA	24	2.27	+	6658.00
L43326	169	30	NA	30	3.35	+	NS
L43358	175	24	NA	24	2.57	+	NS

PC = Post Challenge; PTI = Prior to Infusion; NS = No serum available for testing.
+ = Positive for B. anthracis

Table S-2E: Bacteremia in anthrax-challenged untreated control group

Animal ID	Day 0	24h PC	30h PC	36h PC	42h PC	48h PC	54h PC	60h PC	66h PC	72h PC	84h PC	96h PC	Day 5 PC	Days 7- 36 PC	Terminal
**L49303**	0	0	0	+	+	+	+	+	+	+	+	NA	NA	NA	+
**L49360**	0	0	‡	+	+	+	‡	+	+	+	+	+	+	NA	+
**L49311**	0	0	‡	+	+	+	+	+	+	+	+	NA	NA	NA	+
**L49363**	0	0	+	+	‡	+	+	+	+	NA	NA	NA	NA	NA	+
**L49321**	0	+	+	+	0	+	+	NA	NA	NA	NA	NA	NA	NA	+
**L49375**	0	+	+	+	0	0	+	‡	+	+	+	+	NA	NA	+
**L43316**	0	+	+	+	+	+	+	+	NA	NA	NA	NA	NA	NA	+
**L43352**	0	+	+	+	+	+	NA	NA	NA	NA	NA	NA	NA	NA	+
**L43326**	0	0	+	+	+	‡	+	+	+	+	NA	NA	NA	NA	+
**L43358**	0	+	+	+	+	+	+	NA	NA	NA	NA	NA	NA	NA	+

PC = Post Challenge; 0 = Negative for B. anthracis; ‡ = Positive for B. anthracis with only 1-5 colonies present in the primary streak; + = Positive for B. anthracis; NA- Not applicable due to animal's death

Table S-2F: Proportion of Survivors and Median Time to Death in *B. anthracis*-infected Rabbits Treated with IGIV or ANTHRASIL- Intent-to-treat (ITT) Analysis set

Group	Number Survived/N	Proportion of Survivors (95% Confidence Interval)	Fisher's Exact Test P-value	Median Time to Death in Hours Post-Challenge (95% Confidence Interval)	Log-Rank Test P-value
IGIV	1/50	0.02 (0.00, 0.11)	0.0008*	77.5 (71.2, 85.7)	<0.0001*
ANTHRASIL	13/50	0.26 (0.15, 0.40)		148.5 (113.8, 175.8)	

* = Significant at the 0.05 level; Median time to death was based on Kaplan Meier curve

All animals that have the data needed for the analysis were included (Intent-To-Treat (ITT) group)
