# Supplementary material for: Efficacy of ANTHRASIL (Anthrax Immune Globulin Intravenous (Human)) in rabbit and nonhuman primate models of inhalational anthrax: Data supporting approval under animal rule
Source: PLoS One. 2023 Mar 17;18(3):e0283164. doi: 10.1371/journal.pone.0283164 (PMC10022752; doi:10.1371/journal.pone.0283164)
Supplement: S3 File — (PDF) [file pone.0283164.s003.pdf]

Supplementary data: S-3 (Pharmacokinetics of Anthrasil in the rabbits and NHP)

Figure S-3A: TNA-Time Data (Geometric Mean ± 95% CI; n>3) Following Target IV Infusion of Anthrasil at 15 U/kg in Rabbit Therapeutic Study

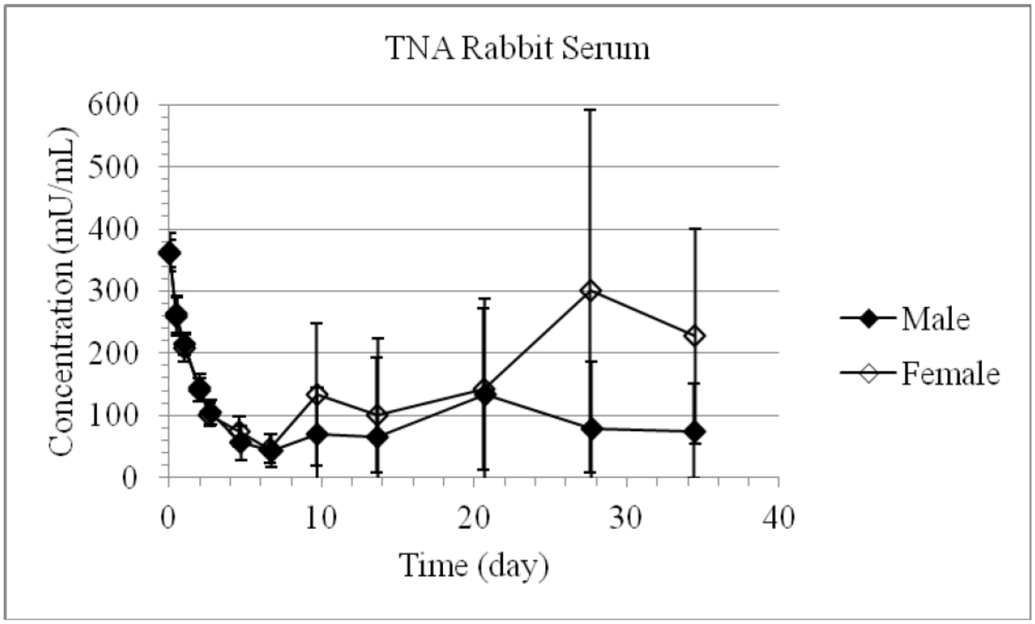

Table S-3A: PK Parameters for Anthrasil (15 U/kg) by TNA in Rabbit Therapeutic Study

Gender	Calculation	C_{max} (mU/mL)	T_{max} (day)	Elimination Rate Constant (day^{-1})	Elimination Half-life (day)	Clearance (mL/day/kg)	Volume of Distribution (mL/kg)	AUC_{0-7day} day*(mU/mL)	AUC_{∞} day*(mU/mL)
Males	N	25	25	10	10	10	10	10	10
	Mean	366	0.0957	0.893	1.11	28.4	33.7	678	710
	SD	81	0.150	0.724	0.69	23.3	9.4	270	307
	SE	16	0.0301	0.229	0.22	7.4	3.0	85	97
	CV%	22.1	157	81.1	62.2	82.0	27.9	39.8	43.2
	Min	171	0.0347	0.245	0.242	12.1	16.0	159	162
	Median	359	0.0417	0.761	0.916	21.2	33.3	689	709
	Max	542	0.500	2.86	2.83	92.3	49.3	1090	1240
	Geometric Mean	357	NA	NA	NA	NA	NA	610	633
Females	N	25	25	12	12	12	12	12	12
	Mean	361	0.0790	1.02	1.26	28.9	36.3	653	693
	SD	53	0.192	0.98	0.80	21.7	13.7	265	297
	SE	11	0.0384	0.28	0.23	6.3	4.0	76	86
	CV%	14.7	243	96.1	63.5	75.1	37.7	40.6	42.9
	Min	238	0.0354	0.302	0.235	12.6	17.0	167	167
	Median	370	0.0417	0.617	1.14	21.0	31.9	677	729
	Max	452	1.00	2.95	2.30	89.7	59.7	1110	1190
	Geometric Mean	357	NA	NA	NA	NA	NA	588	617
Males and Females	N	50	50	22	22	22	22	28	22
	Mean	364	0.0874	0.965	1.19	28.7	35.1	743	701
	SD	68	0.171	0.853	0.74	21.9	11.7	283	294
	SE	10	0.0242	0.182	0.16	4.7	2.5	54	63
	CV%	18.7	196	88.4	62.0	76.3	33.3	38.1	41.9
	Min	171	0.0347	0.245	0.235	12.1	16.0	159	162
	Median	363	0.0417	0.699	0.992	21.2	32.7	759	709
	Max	542	1.00	2.95	2.83	92.3	59.7	1280	1240
	Geometric Mean	357	NA	NA	NA	NA	NA	671	624

a = PK parameters reported to three significant figures; NA = Not applicable; N=Number; SD=Standard deviation; SE=Standard error CV%=Coefficient of variance; Min=Minimum; Max=Maximum

Figure S-3B: ELISA-Time Data (Geometric Mean ± 95% CI; n>3) Following Target IV Infusion of Anthrasil at 15 U/kg in Rabbit Therapeutic Study

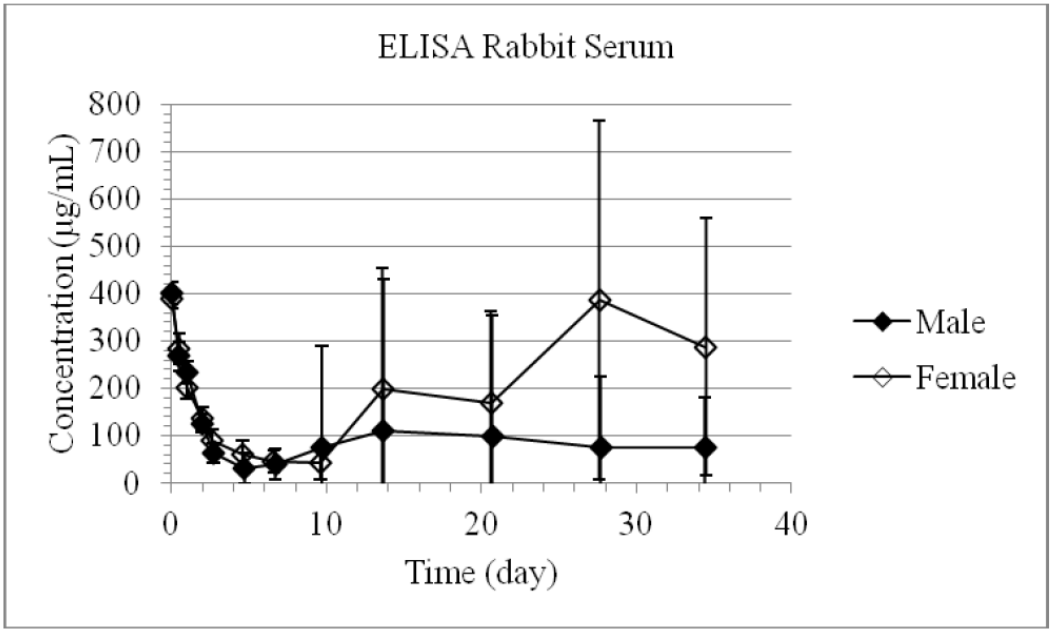

Table S-3B: PK Parameters for Anthrasil (15 U/kg) by anti-PA ELISA in Rabbit Therapeutic Study

Gender	Calculation	C_{max} (µg/mL)	T_{max} (day)	Elimination Rate Constant (day^{-1})	Elimination Half-life (day)	Clearance (mL/day/kg)	Volume of Distribution (mL/kg)	$AUC_{0\text{-}7day}$ (day*µg/mL)	$AUC_{0\text{-}\infty}$ (day*µg/mL)
Males	N	25	25	13	13	13	13	13	13
	Mean	401	0.0591	1.09	0.897	29.7	30.5	611	624
	SD	61	0.0884	0.83	0.550	21.7	12.3	203	213
	SE	12	0.0177	0.23	0.153	6.0	3.4	56	59
	CV%	15.2	150	76.1	61.3	73.1	40.3	33.2	34.1
	Min	287	0.0347	0.288	0.190	16.2	19.8	149	151
	Median	386	0.0417	0.915	0.758	25.2	26.2	590	596
	Max	542	0.483	3.65	2.40	99.6	58.9	918	924
	Geometric Mean	396	NA	NA	NA	NA	NA	566	577
Females	N	25	25	17	17	17	17	17	17
	Mean	389	0.0586	1.18	1.01	28.1	30.3	638	667
	SD	52	0.0899	1.09	0.68	17.3	11.0	242	274
	SE	10	0.0180	0.26	0.17	4.2	2.7	59	66
	CV%	13.4	153	92.4	67.3	61.6	36.3	37.9	41.1
	Min	289	0.0354	0.306	0.160	13.2	12.5	180	182
	Median	377	0.0417	0.930	0.745	23.1	29.3	647	649
	Max	495	0.490	4.32	2.27	82.4	53.8	1070	1140
	Geometric Mean	385	NA	NA	NA	NA	NA	586	605
Males and Females	N	50	50	30	30	30	30	36	30
	Mean	395	0.0588	1.14	0.963	28.8	30.4	690	648
	SD	57	0.0883	0.97	0.622	19.0	11.4	256	246
	SE	8	0.0125	0.18	0.114	3.5	2.1	43	45
	CV%	14.4	150	85.1	64.6	66.0	37.5	37.1	38.0
	Min	287	0.0347	0.288	0.160	13.2	12.5	149	151
	Median	378	0.0417	0.923	0.752	25.1	27.3	686	599
	Max	542	0.490	4.32	2.40	99.6	58.9	1210	1140
	Geometric Mean	391	NA	NA	NA	NA	NA	633	593

a = PK parameters reported to three significant figures; NA = Not applicable; N=Number; SD=Standard deviation; SE=Standard error
CV%=Coefficient of variance; Min=Minimum; Max=Maximum

Table S-3C: PK Parameters for Anthrasil by TNA in NHP Therapeutic Study

Parameter		7.5 U/kg	15 U/kg	30 U/kg
$AUC_{(0-t)}$ (hours*mU/mL)*	N	5	7	9
	Mean	14378.82	23956.92	55594.70
	Geometric Mean	14275.46	22684.97	53314.35
	Std Dev	1829.72	7918.61	15959.13
	Minimum	11235.41	11247.13	27198.49
	Median	14983.71	24910.64	57796.84
	Maximum	15973.26	36304.27	83179.05
	Coeff of Variation	12.73	33.05	28.71
$AUC_{(0-7)}$ (hours*mU/mL)*	N	5	7	9
	Mean	7917.72	13633.67	35029.92
	Geometric Mean	7715.39	13143.26	33633.94
	Std Dev	2038.91	3646.53	10606.23
	Minimum	5590.47	7238.70	20281.36
	Median	7692.80	14482.78	35162.81
	Maximum	10988.00	18464.99	51633.68
	Coeff of Variation	25.75	26.75	30.28
$C_{(max)}$ (mU/mL)	N	14	14	14
	Mean	142.88	247.57	515.92
	Geometric Mean	139.25	234.67	501.45
	Std Dev	34.60	75.32	135.10
	Minimum	98.30	87.20	386.30
	Median	136.75	246.30	508.20
	Maximum	218.60	392.00	795.30
	Coeff of Variation	24.22	30.42	26.19
T_{max} (hours) **	N	14	14	14
	Mean	0.21	4.05	10.70
	Std Dev	0.19	6.46	7.91
	Minimum	0.07	0.05	2.52
	Median	0.14	0.12	9.64
	Maximum	0.68	17.53	32.27
	Coeff of Variation	87.33	159.80	73.93

^ Information for calculation of PK parameters in cut off by day 14.

* Animals with short follow-up, which is less than 7 days (half of the eligible PK evaluation period, are considered not calculable for the AUC calculation, and are excluded from the summary.

** Actual time post infusion.

Figure S3-C: Mean plot of TNA concentration in NHP therapeutic study

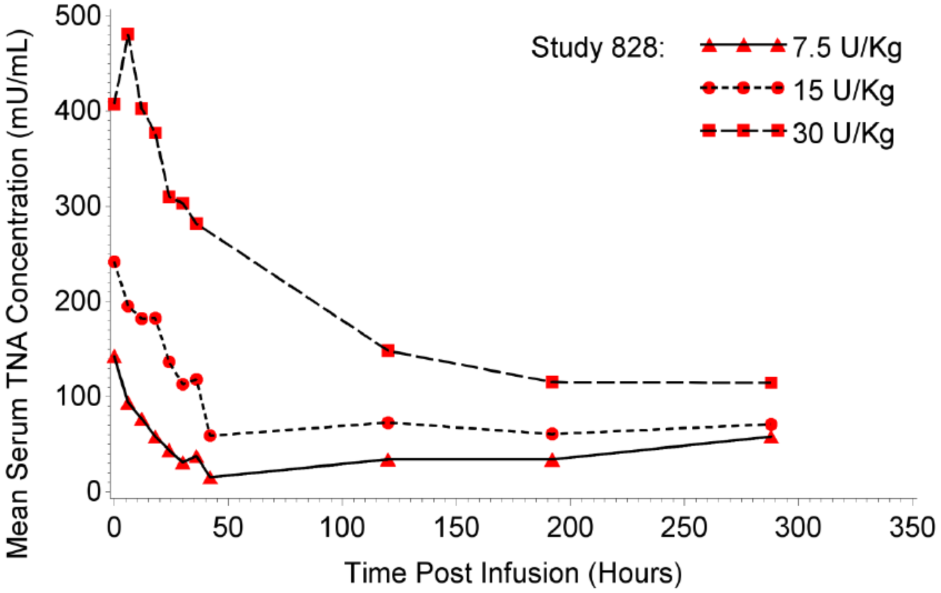

In this study, samples were collected from surviving animals through to Day 28 post-challenge. However, many of the animals that survived past Day 14 (336 hr) post challenge exhibited a rebound of anti-PA levels, indicating a humoral immune effect in response to B. anthracis infection. This humoral immune effect makes the measurement of circulating AIGIV levels unreliable via the TNA assay. Therefore, the PK analysis of ANTHRASIL (AIGIV) was only performed through day 14 post challenge. As such AUC0-14 is assigned as AUC0-t accordingly in this report. Due to this cut-off on 14 days, it is impossible to determine a reliable estimate for the elimination rate constant (Kel). Consequently, the PK parameters related to Kel (e.g. AUC0-∞, Kel, T½, Cl and Vd) are not calculated for this study.

The nominal time post-infusion for study is defined as every time point 6 hours post-infusion for the first 72 hours. The time points after 72 hours are reviewed by statistician and binned according to the original day 7, day 10 and day 14 post challenge visit with ±1 day visit window. Because most monkeys became positive for toxemia at 30-48 h post anthrax challenge, the nominal time points after 72 hours were binned to be day 5, day 8 and day 12 post-infusion to normalize this variability.

Table S3-D: PK Parameters for Anthrasil (TNA) by Gender in NHP Therapeutic Study

Parameter		Male			Female		
		7.5 U/kg	15 U/kg	30 U/kg	7.5 U/kg	15 U/kg	30 U/kg
AUC(0-t)* (hours*mU/mL)	N	4	4	4	1	3	5
	Mean	13980.20	27593.49	65133.92	15973.26	19108.17	47963.33
	Geometric Mean	13879.99	26870.26	64172.61	15973.26	18100.62	45966.32
	Std Dev	1845.15	7143.63	13249.25	-	7060.54	14626.59
	Minimum	11235.41	18811.04	52265.27	15973.26	11247.13	27198.49
	Median	14777.30	27629.32	62545.68	15973.26	21166.75	47422.02
	Maximum	15130.81	36304.27	83179.05	15973.26	24910.64	65140.11
	Coeff of Variation	13.20	25.89	20.34	-	36.95	30.50
AUC(0-7)* (hours*mU/mL)	N	4	4	4	1	3	5
	Mean	7758.80	15386.07	41381.29	8553.42	11297.12	29948.83
	Geometric Mean	7519.04	15230.17	40112.24	8553.42	10798.61	29213.15
	Std Dev	2318.29	2526.35	11535.56	-	3998.36	7250.57
	Minimum	5590.47	12493.10	28244.76	8553.42	7238.70	20281.36
	Median	7228.37	15293.10	42823.36	8553.42	11420.09	27824.37
	Maximum	10988.00	18464.99	51633.68	8553.42	15232.58	37892.57
	Coeff of Variation	29.88	16.42	27.88	-	35.39	24.21
Cmax (mU/mL)	N	7	8	7	7	6	7
	Mean	137.51	272.58	555.86	148.24	214.23	475.99
	Geometric Mean	135.99	266.47	533.45	142.59	198.09	471.37
	Std Dev	21.15	64.20	175.53	45.60	81.52	70.80
	Minimum	98.30	201.70	390.90	102.70	87.20	386.30
	Median	139.00	247.10	541.80	129.80	226.05	479.50
	Maximum	166.10	392.00	795.30	218.60	305.70	551.50
	Coeff of Variation	15.38	23.55	31.58	30.76	38.05	14.87
Tmax** (hours)	N	7	8	7	7	6	7
	Mean	0.29	4.22	13.60	0.14	3.82	7.81
	Std Dev	0.24	6.52	9.93	0.03	7.00	4.19
	Minimum	0.07	0.10	2.52	0.08	0.05	3.08
	Median	0.22	0.13	15.10	0.13	0.11	9.53
	Maximum	0.68	17.12	32.27	0.17	17.53	14.52
	Coeff of Variation	85.00	154.60	73.04	19.32	183.49	53.66
