# Supplementary material for: Efficacy of ANTHRASIL (Anthrax Immune Globulin Intravenous (Human)) in rabbit and nonhuman primate models of inhalational anthrax: Data supporting approval under animal rule
Source: PLoS One. 2023 Mar 17;18(3):e0283164. doi: 10.1371/journal.pone.0283164 (PMC10022752; doi:10.1371/journal.pone.0283164)
Supplement: S4 File — (PDF) [file pone.0283164.s004.pdf]

Supplementary data: S-4 (Additional data from NHP therapeutic study)

Table S-4A: Experimental design of Nonhuman Primate Therapeutic Study

Group	No. of animals	Treatment	Treatment Dose (TNA U/kg)	Treatment time
1	16	IGIV	N/A	Onset of toxemia
2	16	ANTHRASIL	7.5	
3	16	ANTHRASIL	15.0	
4	16	ANTHRASIL	30.0	

Table S-4B: Proportion of Animals (Nonhuman primate) in each Group with Abnormal White Blood Cell Counts Post-treatment by Study Time

Study Time	Bin	Group	No. Abnormal/N #	Proportion Abnormal (95% Confidence Interval)
Treatment to 6 Hours Post-Treatment	1	1	NA	NA
		2	5/16	0.31 (0.11, 0.59)
		3	2/16	0.13 (0.02, 0.38)
		4	NA	NA
6 to 12 Hours Post-Treatment	2	1	1/16	0.06 (0.00, 0.30)
		2	3/15	0.20 (0.04, 0.48)
		3	3/16	0.19 (0.04, 0.46)
		4	1/16	0.06 (0.00, 0.30)
12 to 18 Hours Post-Treatment	3	1	1/15	0.07 (0.00, 0.32)
		2	6/15	0.40 (0.16, 0.68)
		3	7/16	0.44 (0.20, 0.70)
		4	3/16	0.19 (0.04, 0.46)
18 to 24 Hours Post-Treatment	4	1	3/12	0.25 (0.05, 0.57)
		2	7/13	0.54 (0.25, 0.81)
		3	8/14	0.57 (0.29, 0.82)
		4	1/15	0.07 (0.00, 0.32)
24 to 30 Hours Post-Treatment	5	1	4/10	0.40 (0.12, 0.74)
		2	2/13	0.15 (0.02, 0.45)
		3	3/12	0.25 (0.05, 0.57)
		4	0/13	0.00 (0.00, 0.25)
30 to 36 Hours Post-Treatment	6	1	4/7	0.57 (0.18, 0.90)
		2	2/12	0.17 (0.02, 0.48)
		3	1/8	0.13 (0.00, 0.53)
		4	1/13	0.08 (0.00, 0.36)
36 to 42 Hours Post-Treatment	7	1	2/4	0.50 (0.07, 0.93)
		2	1/7	0.14 (0.00, 0.58)
		3	1/5	0.20 (0.01, 0.72)
		4	0/8	0.00 (0.00, 0.37)
42 to 48 Hours Post-Treatment	8	1	--	--
		2	0/2	0.00 (0.00, 0.84)
		3	1/1	1.00 (0.03, 1.00)
		4	--	--

\# Number of animals alive at study time.

NA Time points are missing since all animals in these groups were still receiving treatment at scheduled blood draw.

-- No animals available in this group at this bin.

Table S-4C: Proportion of Animals (Nonhuman primate) in each Group with Abnormal Neutrophil Cell Counts Post-treatment by Study Time

Study Time	Bin	Group	No. Abnormal/N #	Proportion Abnormal (95% Confidence Interval)
Treatment to 6 Hours Post-Treatment	1	1	NA	NA
		2	3/16	0.19 (0.04, 0.46)
		3	4/16	0.25 (0.07, 0.52)
		4	NA	NA
6 to 12 Hours Post-Treatment	2	1	3/16	0.19 (0.04, 0.46)
		2	5/15	0.33 (0.12, 0.62)
		3	4/16	0.25 (0.07, 0.52)
		4	5/16	0.31 (0.11, 0.59)
12 to 18 Hours Post-Treatment	3	1	6/15	0.40 (0.16, 0.68)
		2	11/15	0.73 (0.45, 0.92)
		3	10/16	0.63 (0.35, 0.85)
		4	9/16	0.56 (0.30, 0.80)
18 to 24 Hours Post-Treatment	4	1	6/12	0.50 (0.21, 0.79)
		2	7/13	0.54 (0.25, 0.81)
		3	10/14	0.71 (0.42, 0.92)
		4	7/15	0.47 (0.21, 0.73)
24 to 30 Hours Post-Treatment	5	1	5/10	0.50 (0.19, 0.81)
		2	5/13	0.38 (0.14, 0.68)
		3	4/12	0.33 (0.10, 0.65)
		4	2/13	0.15 (0.02, 0.45)
30 to 36 Hours Post-Treatment	6	1	4/7	0.57 (0.18, 0.90)
		2	1/12	0.08 (0.00, 0.38)
		3	1/8	0.13 (0.00, 0.53)
		4	3/13	0.23 (0.05, 0.54)
36 to 42 Hours Post-Treatment	7	1	2/4	0.50 (0.07, 0.93)
		2	2/7	0.29 (0.04, 0.71)
		3	1/5	0.20 (0.01, 0.72)
		4	1/8	0.13 (0.00, 0.53)
42 to 48 Hours Post-Treatment	8	1	--	--
		2	1/2	0.50 (0.01, 0.99)
		3	0/1	0.00 (0.00, 0.98)
		4	--	--

\# Number of animals alive at study time.

NA Time points are missing since all animals in these groups were still receiving treatment at scheduled blood draw.

-- No animals available in this group at this bin.

Table S-4D: Proportion of Animals (Nonhuman primate) in each Group with Abnormal Lymphocyte Cell Counts Post-treatment by Study Time

Study Time	Bin	Group	No. Abnormal/N #	Proportion Abnormal (95% Confidence Interval)
Treatment to 6 Hours Post-Treatment	1	1	NA	NA
		2	0/16	0.00 (0.00, 0.21)
		3	1/16	0.06 (0.00, 0.30)
		4	NA	NA
6 to 12 Hours Post-Treatment	2	1	3/16	0.19 (0.04, 0.46)
		2	5/15	0.33 (0.12, 0.62)
		3	6/16	0.38 (0.15, 0.65)
		4	9/16	0.56 (0.30, 0.80)
12 to 18 Hours Post-Treatment	3	1	3/15	0.20 (0.04, 0.48)
		2	4/15	0.27 (0.08, 0.55)
		3	6/16	0.38 (0.15, 0.65)
		4	5/16	0.31 (0.11, 0.59)
18 to 24 Hours Post-Treatment	4	1	1/12	0.08 (0.00, 0.38)
		2	2/13	0.15 (0.02, 0.45)
		3	2/14	0.14 (0.02, 0.43)
		4	4/15	0.27 (0.08, 0.55)
24 to 30 Hours Post-Treatment	5	1	1/10	0.10 (0.00, 0.45)
		2	3/13	0.23 (0.05, 0.54)
		3	0/12	0.00 (0.00, 0.26)
		4	0/13	0.00 (0.00, 0.25)
30 to 36 Hours Post-Treatment	6	1	0/7	0.00 (0.00, 0.41)
		2	1/12	0.08 (0.00, 0.38)
		3	1/8	0.13 (0.00, 0.53)
		4	1/13	0.08 (0.00, 0.36)
36 to 42 Hours Post-Treatment	7	1	2/4	0.50 (0.07, 0.93)
		2	1/7	0.14 (0.00, 0.58)
		3	1/5	0.20 (0.01, 0.72)
		4	0/8	0.00 (0.00, 0.37)
42 to 48 Hours Post-Treatment	8	1	--	--
		2	0/2	0.00 (0.00, 0.84)
		3	0/1	0.00 (0.00, 0.98)
		4	--	--

Number of animals alive at study time.

NA Time points are missing since all animals in these groups were still receiving treatment at scheduled blood draw.

-- No animals available in this group at this bin.

Table S-4E: Proportion of Animals (Nonhuman primate) in each Group with Abnormal Neutrophil/Lymphocyte Ratio Post-treatment by Study Time

Study Time	Bin	Group	No. Abnormal/N #	Proportion Abnormal (95% Confidence Interval)
Treatment to 6 Hours Post-Treatment	1	1	NA	NA
		2	0/16	0.00 (0.00, 0.21)
		3	1/16	0.06 (0.00, 0.30)
		4	NA	NA
6 to 12 Hours Post-Treatment	2	1	4/16	0.25 (0.07, 0.52)
		2	9/15	0.60 (0.32, 0.84)
		3	8/16	0.50 (0.25, 0.75)
		4	10/16	0.63 (0.35, 0.85)
12 to 18 Hours Post-Treatment	3	1	7/15	0.47 (0.21, 0.73)
		2	10/15	0.67 (0.38, 0.88)
		3	12/16	0.75 (0.48, 0.93)
		4	11/16	0.69 (0.41, 0.89)
18 to 24 Hours Post-Treatment	4	1	3/12	0.25 (0.05, 0.57)
		2	8/13	0.62 (0.32, 0.86)
		3	9/14	0.64 (0.35, 0.87)
		4	9/15	0.60 (0.32, 0.84)
24 to 30 Hours Post-Treatment	5	1	3/10	0.30 (0.07, 0.65)
		2	6/13	0.46 (0.19, 0.75)
		3	4/12	0.33 (0.10, 0.65)
		4	2/13	0.15 (0.02, 0.45)
30 to 36 Hours Post-Treatment	6	1	3/7	0.43 (0.10, 0.82)
		2	1/12	0.08 (0.00, 0.38)
		3	1/8	0.13 (0.00, 0.53)
		4	4/13	0.31 (0.09, 0.61)
36 to 42 Hours Post-Treatment	7	1	2/4	0.50 (0.07, 0.93)
		2	2/7	0.29 (0.04, 0.71)
		3	2/5	0.40 (0.05, 0.85)
		4	1/8	0.13 (0.00, 0.53)
42 to 48 Hours Post-Treatment	8	1	--	--
		2	1/2	0.50 (0.01, 0.99)
		3	0/1	0.00 (0.00, 0.98)
		4	--	--

Number of animals alive at study time.
NA Time points are missing since all animals in these groups were still receiving treatment at scheduled blood draw.
-- No animals available in this group at this bin.

Table S-4F: Proportion of Animals in Each Group Abnormal Prior To Treatment for CRP, Bacteremia, PA-ECL, and qPCR Data

Parameter	Group	No. Abnormal/N #	Proportion Abnormal (95% Confidence Interval)
C-Reactive Protein	1	12/16	0.75 (0.48, 0.93)
	2	11/16	0.69 (0.41, 0.89)
	3	9/16	0.56 (0.30, 0.80)
	4	10/16	0.63 (0.35, 0.85)
Bacteremia	1	11/16	0.69 (0.41, 0.89)
	2	11/16	0.69 (0.41, 0.89)
	3	14/16	0.88 (0.62, 0.98)
	4	10/16	0.63 (0.35, 0.85)
PA-ECL	1	16/16	1.00 (0.79, 1.00)
	2	16/16	1.00 (0.79, 1.00)
	3	16/16	1.00 (0.79, 1.00)
	4	14/16	0.88 (0.62, 0.98)
qPCR	1	12/16	0.75 (0.48, 0.93)
	2	7/16	0.44 (0.20, 0.70)
	3	8/16	0.50 (0.25, 0.75)
	4	9/16	0.56 (0.30, 0.80)

Total number of animals in each group.

Table S-4G: Proportion of Animals in Each Group Abnormal Prior To Treatment for Hematology, Temperature and Activity Parameters

Parameter	Group	No. Abnormal/N #	Proportion Abnormal (95% Confidence Interval)
WBC	1	1/16	0.06 (0.00, 0.30)
	2	6/16	0.38 (0.15, 0.65)
	3	3/16	0.19 (0.04, 0.46)
	4	2/16	0.13 (0.02, 0.38)
Neutrophils	1	0/16	0.00 (0.00, 0.21)
	2	1/16	0.06 (0.00, 0.30)
	3	0/16	0.00 (0.00, 0.21)
	4	2/16	0.13 (0.02, 0.38)
Lymphocytes	1	4/16	0.25 (0.07, 0.52)
	2	7/16	0.44 (0.20, 0.70)
	3	5/16	0.31 (0.11, 0.59)
	4	4/16	0.25 (0.07, 0.52)
NL Ratio	1	4/16	0.25 (0.07, 0.52)
	2	2/16	0.13 (0.02, 0.38)
	3	4/16	0.25 (0.07, 0.52)
	4	2/16	0.13 (0.02, 0.38)
SIBT6	1	6/16	0.38 (0.15, 0.65)
	2	6/16	0.38 (0.15, 0.65)
	3	6/16	0.38 (0.15, 0.65)
	4	4/16	0.25 (0.07, 0.52)
Activity	1	0/16	0.00 (0.00, 0.21)
	2	0/16	0.00 (0.00, 0.21)
	3	2/16	0.13 (0.02, 0.38)
	4	0/16	0.00 (0.00, 0.21)

Total number of animals in each group.
